# How absorber chemistry shapes sustainability, stability and end-of-life options in silicon, CdTe and perovskite photovoltaics

**DOI:** 10.1039/d6sc03126a

**Published:** 2026-10-13

**Authors:** Karen Valadez-Villalobos, Rodrigo García-Rodríguez, Rhys Charles, Matthew L. Davies

**Affiliations:** a SPECIFIC IKC, Materials Science and Engineering, Faculty of Science and Engineering, Swansea University Swansea UK m.l.davies@swansea.ac.uk; b School of Chemistry and Physics, University of KwaZulu-Natal Durban South Africa

## Abstract

The sustainability of photovoltaic technologies cannot be assessed independently of their absorber chemistry. In this perspective, we compare crystalline silicon (c-Si), cadmium telluride (CdTe) and halide perovskites, tracing how their fundamental optoelectronic and chemical properties shape material demand, manufacturing, operational stability and end-of-life pathways. Silicon has an indirect bandgap and limited defect tolerance, which necessitate thick wafers, ultra-high-purity feedstocks and energy- and capital-intensive processing. Its chemically robust and insoluble structure supports long operational lifetimes but complicates material recovery. By contrast, the strong absorption and greater defect tolerance of CdTe and halide perovskites enable thin-film architectures, lower absorber-material requirements and potentially less intensive manufacturing and recovery processes. These advantages are accompanied by distinct challenges, including tellurium criticality and cadmium toxicity for CdTe, and limited stability and lead management for perovskites. No absorber choice is intrinsically sustainable: progress requires the coordinated optimisation of performance, durability, resource availability, manufacturing and circularity across the complete photovoltaic lifecycle.

## Abstract 2

Absorber chemistry links atomic-scale properties to lifecycle outcomes, governing manufacturing, stability and circularity in silicon, CdTe and perovskite photovoltaics.

## Graphical abstract

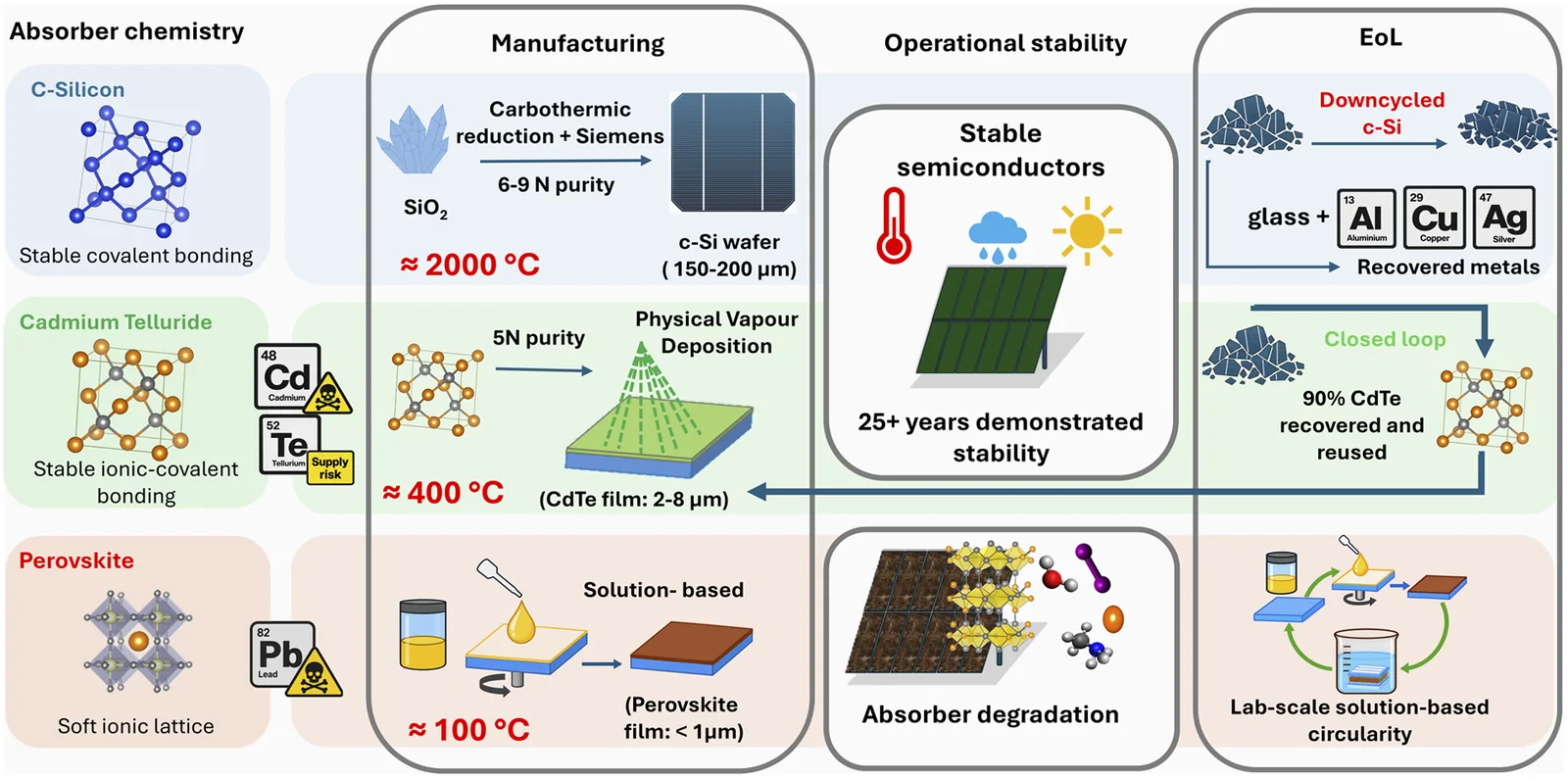

## 1 Introduction

Photovoltaic (PV) deployment is expanding at an unprecedented scale, requiring significant consideration of PV sustainability. In 2024, ∼600 GW of new PV capacity was commissioned, increasing cumulative installations to >2.2 TW and raising PV's contribution to global electricity consumption above 10% for the first time.^1^ As deployment moves into the terawatt era, the sustainability of PV technologies increasingly depends not only on conversion efficiency and cost, but also on material availability, manufacturing requirements, operational durability and the management of modules at end of life (EoL). These themes are deeply interconnected and fundamentally rooted in the chemistry of the absorber materials. In turn, these characteristics influence the quantity and purity of material required, the energy and complexity of manufacturing, achievable operational lifetime, environmental risks and the feasibility of material recovery or device remanufacture. Absorber chemistry therefore provides a unifying framework for understanding the sustainability consequences of PV technology choices.

In this perspective, we apply this framework to three technologically important but chemically distinct absorber systems: crystalline silicon (c-Si), cadmium telluride (CdTe) and halide perovskites. CdTe is retained as the representative commercial thin film because comparable industrial manufacturing, warranty and EoL data are not available for CIGS following the contraction of commercial production after Solar Frontier's 2022 exit.^2^ The comparison is therefore defined by the availability of industrial-scale lifecycle evidence rather than by an attempt to represent every PV absorber class. Together, they encompass the dominant commercial technology, the only inorganic thin-film technology currently manufactured at substantial volume, and a rapidly developing emerging technology.

The aim is to help researchers and technology developers assess the sustainability implications of PV design choices and to highlight key areas where chemical science can drive innovation for a circular energy future. We trace how intrinsic and interfacial properties create different advantages, constraints and trade-offs across materials supply, manufacturing, stability and EoL management. Attention is given to the distinction between absorber-level properties and module-level outcomes: contacts, transparent conductors, encapsulants and glass can dominate mass, energy demand, cost or failure even when their requirements originate partly in the absorber. By connecting these levels, the analysis identifies where chemical science can reduce environmental burdens, improve circularity and avoid displacing sustainability challenges from one lifecycle stage to another.

## 2 Materials

Absorber chemistry establishes many of the initial conditions governing PV performance and sustainability. The electronic structure determines the usable portion of the solar spectrum, optical absorption and achievable photovoltage, while bonding and defect physics influence carrier transport, purity requirements and processing tolerance. Together with density and elemental composition, these properties determine absorber thickness, material demand and aspects of manufacturing complexity. They also influence degradation pathways, environmental hazards and the chemical forms in which constituent materials can be recovered at EoL. Crystalline silicon, CdTe and halide perovskites illustrate markedly different combinations of these properties. Silicon combines high efficiency and long operational lifetime with comparatively weak near-band-edge absorption, stringent impurity control and energy-intensive feedstock production. CdTe provides strong absorption in a micrometre-scale polycrystalline film but depends on tellurium, a comparatively scarce element, and requires responsible management of cadmium. Halide perovskites achieve strong absorption and high efficiencies in sub-micrometre polycrystalline films processed from relatively low-purity precursors, but their ionic character contributes to chemical and operational instability and complicates the containment and recovery of lead. These differences create lifecycle trade-offs rather than a simple hierarchy of sustainability, as will be explored further in the following sections.

### 2.1 Shockley–Queisser limit

The maximum theoretical efficiency of a single-junction solar cell is defined by the Shockley–Queisser (SQ) limit, which represents the balance between photon absorption and radiative recombination in an ideal semiconductor absorber.^3,4^ The limit arises because only photons with energies greater than the semiconductor bandgap can generate electron–hole pairs, while the excess energy of the excited carrier is transferred to the crystal lattice as heat through phonon interactions, a process that occurs on femtosecond timescales, far faster than charge extraction can occur.

Consequently, materials with smaller bandgaps absorb a wider range of the solar spectrum but suffer greater thermalisation losses, whereas larger-bandgap materials absorb a narrower range but generate higher voltage. This trade-off leads to an optimal single-junction bandgap of approximately 1.3–1.4 eV, corresponding to a maximum efficiency of *ca.* 33% under AM1.5G illumination.^4,5^ For crystalline silicon, with a bandgap of 1.12 eV, the SQ limit is *ca.* 32.2%, with commercial monocrystalline silicon devices reaching efficiencies of 24%, and lab-scale devices achieving efficiencies over 27%, representing ≈84% of the theoretical limit.^2^ For CdTe, with a bandgap of approximately 1.45–1.5 eV, close to the SQ optimum, the theoretical single-junction limit exceeds 32%; current lab-scale records stand at 23.2%, with commercial module efficiencies in the range of 17–20%, representing an SQ utilisation of ≈73%.^2,6,7^ For hybrid perovskites, the theoretical limit is approximately 31% for a bandgap of 1.55 eV.^8^ Lab-scale single-junction devices have reached a certified efficiency of 27.3%,^6^ corresponding to ≈88% of the detailed-balance limit at that bandgap, up from roughly 65% a decade ago. All the SQ utilisation values given here are based on record laboratory scale efficiencies for fair comparison.

### 2.2 Direct *vs.* indirect bandgap

In an indirect-bandgap semiconductor such as silicon, optical excitation near the band edge generally requires both a photon and a lattice vibration to conserve momentum (Fig. 1a). Absorption is consequently weaker near the band edge than in direct-bandgap semiconductors. Silicon has an absorption coefficient of approximately 10^2^–10^3^ cm^−1^ close to its band edge, compared with values approaching 10^5^ cm^−1^ over relevant portions of the visible spectrum for CdTe and lead-halide perovskites (Fig. 1b).^9–12^ Commercial silicon devices therefore use wafers approximately 150–200 µm thick together with surface texturing and rear reflectors that increase optical path length. Thickness is not determined by absorption alone: mechanical handling, breakage, kerf loss and established production equipment constrain how thin wafers can become. Nevertheless, the indirect bandgap is a fundamental reason why the silicon absorber remains much thicker than those used in direct-bandgap thin-film technologies.

**Fig. 1:**
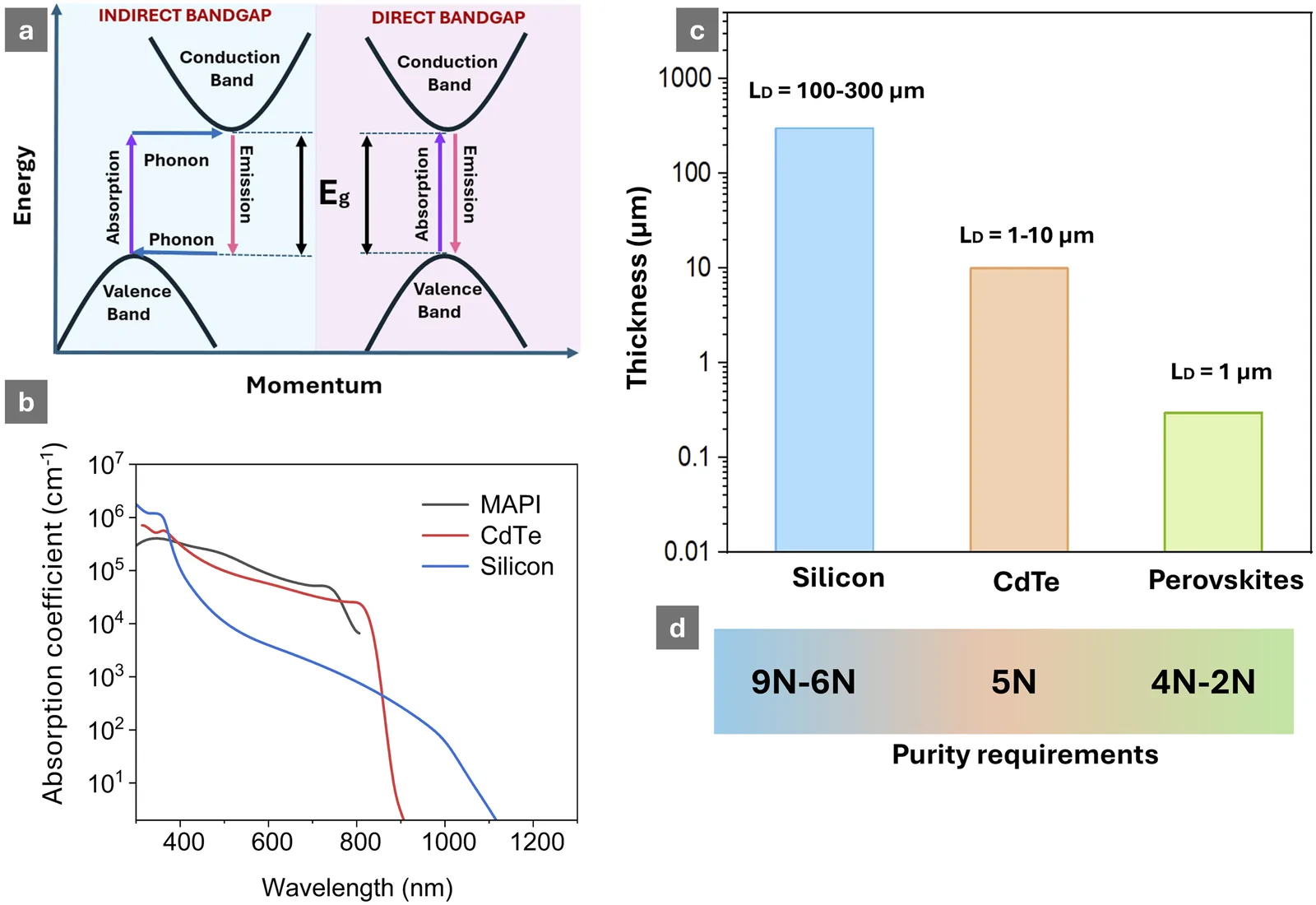
Material properties governing absorber thickness, carrier collection, and purity requirements in crystalline silicon, CdTe, and halide perovskite solar cells. (a) Schematic band diagrams for indirect and direct bandgap semiconductors. (b) Absorption coefficient as a function of wavelength for CH_3_NH_3_PbI_3_, CdTe, and crystalline silicon (adapted from ref. 12). (c) Absorber thickness ranges and typical minority carrier diffusion lengths (*L*_D_) for each technology. (d) Purity requirements reflecting the dependence of carrier lifetime and diffusion length on defect tolerance across the three absorber materials.

CdTe and most photovoltaic lead-halide perovskites behave as direct or near-direct-bandgap absorbers, meaning photon absorption requires only a single interaction with no phonon participation. Their high absorption coefficients of 10^4^–10^5^ cm^−1^ allow near-complete light absorption in much thinner layers (Fig. 1c).^10,13,14^ Current commercial CdTe absorbers are approximately 3 µm thick, within a broader 2–8 µm range across device generations, whereas perovskite absorber layers are commonly 300–700 nm thick.^13–15^ Reduced thickness lowers the semiconductor mass in the device, but not necessarily total module mass, where glass, encapsulation, contacts and transparent conductors can dominate.

### 2.3 Carrier lifetime, diffusion length and purity

Photogenerated carriers must reach charge-selective interfaces before recombining. Carrier lifetime describes the average time before recombination and is determined by the density of defects and impurities and their effectiveness as recombination centres. The distance a carrier can travel within this lifetime is its diffusion length (*L*_D_), which must be comparable to or exceed the distance carriers must travel to reach the collecting junction. In thick absorbers with little internal field, such as silicon, this approximates the absorber thickness, whereas in thin, lightly doped devices the built-in field can extend across much of the layer, so drift assists collection and relaxes this requirement.^16^ The defect and impurity tolerance of the absorber therefore determines the purity requirements to achieve sufficient diffusion length, effective charge collection and, ultimately, good device performance.^16^

Silicon's rigid covalent bonding means that any impurity or lattice distortion tends to introduce deep electronic levels within the bandgap.^3,17^ These act as traps that sequentially capture an electron and a hole, allowing them to recombine non-radiatively through the Shockley–Read–Hall (SRH) mechanism, without contributing to photocurrent.^18^ Transition metals are particularly harmful in this regard, introducing mid-gap trap states that capture both electrons and holes. Even trace concentrations introduced *via* the feedstock during crystal growth or during cell processing can, therefore, significantly reduce carrier lifetime and limit device performance.^19–23^ Since the minority carrier diffusion length must exceed the absorber thickness for efficient carrier collection, and silicon absorbers are typically 150–200 µm thick, diffusion lengths of several hundred microns are required in high-quality material. Given the sensitivity of carrier lifetime to metallic impurities acting as SRH recombination centres, maintaining such diffusion lengths demands that impurity concentrations be kept to extremely low levels, driving solar-grade silicon to purities of 6 N (99.9999%) or above (Fig. 1d).^18^

In polycrystalline CdTe, grain boundaries concentrate defect states and represent the primary limitation on carrier lifetime. A post-deposition treatment with CdCl_2_ addresses this directly: chlorine diffuses preferentially along grain boundaries and accumulates there, electrically passivating recombination-active sites while simultaneously promoting grain growth.^24^ The result is a suppression of non-radiative recombination throughout the film, enabling longer carrier lifetimes sufficient to span the absorber thickness and support efficient carrier collection.^25^ CdTe's tolerance to grain boundary defects, when effectively passivated, means that bulk material purity requirements are less stringent than for silicon, with 5 N (99.999%) purity generally considered sufficient for device-grade material.^26,27^

Halide perovskites achieve diffusion lengths exceeding 1 µm without requiring bulk passivation, more than the typical absorber thickness of 300–700 nm.^28^ This stems from their remarkable defect tolerance, which is rooted in the electronic structure of the material. In the ABX_3_ perovskite structure, where A is typically an organic or inorganic cation, B is a divalent metal such as lead, and X is a halide such as iodide, the valence band maximum (VBM) derives from antibonding hybridisation between the Pb 6s lone pair and halide np orbitals, while the conduction band minimum (CBM) is dominated by spin–orbit-split Pb 6p states; this raises the VBM and lowers the CBM to the point that the states introduced by common native defects fall at or near the band edges rather than in the gap.^29^ Unlike deep traps present in the silicon absorber,^30^ which efficiently capture and recombine carriers *via* the SRH mechanism, shallow states release carriers back to the bands before recombination can occur and therefore do not strongly limit carrier lifetime. High efficiencies are consequently achievable even in polycrystalline films with significant defect densities. That is the core of defect tolerance across hybrid and inorganic lead-halide perovskites.^31^

Consequently, purity requirements for perovskite absorbers are less demanding than those for silicon and CdTe, with high efficiencies achieved using precursors in the 99–99.9% purity range.^32^ While higher precursor purity has been correlated with improved performance and stability, the purification itself is achieved through wet chemistry salt synthesis approaches that are intrinsically less energy-intensive than the elemental purification processes required for silicon.^33–35^

While purity requirements may be lower, precursor stoichiometry, coordination chemistry, solvent composition, crystallisation pathway, humidity and batch variability can be more important than nominal purity alone. The most relevant defects affecting the performance of perovskites arise from how the film assembles during solution processing, a process governed by precursor coordination chemistry and crystallisation kinetics rather than elemental purity. The strong Lewis acidity of Pb^2+^ drives coordination with iodide and solvent molecules, producing a distribution of iodoplumbate complexes, [PbI_2+*n*_]^*n*−^ (*n* = 1–4), whose relative populations govern the crystallisation pathway and resulting structural defect density,^36^ with higher-coordination species favouring more homogeneous nucleation and complete conversion^37,38^*versus* under-coordinated Pb species seeding incomplete conversion, residual PbI_2_, and pinhole formation.^39^ In mixed-halide and mixed-cation formulations, coordination preference varies with ionic species and with the solvent used, multiplying the number of complexes and intermediate phases that can coexist and affecting crystal orientation and phase segregation.^40–42^

Beyond the composition of the perovskite precursor solution, other variables during the processing of the absorber such as antisolvent choice, volume, and dripping timing constitute a further source of variation, affecting intermediate phases, residual strain, and grain uniformity.^43–46^ The local atmosphere influences film coverage and crystallisation.^47^ Moderate humidity has been shown to improve material properties for MAPbI_3_ if applied at the optimised stage,^48^ whereas, for formamidinium-based perovskites, uncontrolled humidity generates variable intermediate phases and non-uniform films.^47–49^ The large variation in these interacting variables propagates into heterogeneity in composition, phase, grain structure, and strain across multiple length scales.^50,51^ Because the local bandgap, defect density, and non-radiative recombination rate are set directly by the local structure and composition, heterogeneity in composition and morphology across multiple scales causes measurable spatial variation in optoelectronic properties: from grain-to-grain differences in photoluminescence and carrier lifetime^52^ to nanoscale chemical heterogeneity dominating the local optoelectronic response independent of average composition,^53^ to halide segregation splitting a single film into distinct bandgap domains.^54^

Mobile ionic species add a further dynamic component under operational conditions: ions redistribute reversibly under the applied field, producing short-term *J*–*V* hysteresis, while, over longer timescales, the same migration instead drives partially reversible losses that track light/dark cycling and, if left unrecovered, can compound into irreversible degradation.^55,56^ These challenges are largely specific to perovskites. As a stable single-phase binary compound and a single-element covalent crystal, respectively, CdTe and silicon are intrinsically far less susceptible to compositional and phase-level variability. The two material classes therefore present opposite manufacturing landscapes: silicon and CdTe demand energy-intensive purification but yield structurally simple, reproducible absorbers, whereas the low-energy solution processing of perovskites poses challenges for reproducibility and stability.

### 2.4 Material intensity and device-stack context

Absorber thickness, density, stoichiometry, module efficiency and manufacturing yield together determine feedstock demand per unit of deployed power. Table 1 separates absorber intensity from the rest of the device stack. Silicon requires substantially more absorber mass because the wafer is orders of magnitude thicker than a thin film. Geological abundance does not remove the energy burden of quartz reduction, purification, crystallisation and wafering, while kerf represents the loss of material that has already acquired high embodied energy. CdTe uses far less semiconductor mass, but tellurium supply is constrained by scarcity and by-product production. Selenium can be incorporated into modern CdSe_*x*_Te_1−*x*_ absorbers. Perovskites use small absorber masses, but lead toxicity remains relevant independently of total mass.

**Table 1:** Device structure, absorber-material intensity and degradation characteristics of crystalline silicon, CdTe and halide-perovskite photovoltaics. Material intensities assume the compositions, layer thicknesses and module efficiencies stated in the SI (S1)^a^

	Absorber material		
	Silicon	CdTe	Perovskites
Structure	Thick (∼150–200 µm) Si wafer with a p–n junction, surface passivation layers, and front/back metallisation, typically encapsulated between glass sheets with EVA.	Thin-film stack: TCO, buffer layer (CdS or MgZnO), CdSe_*x*_Te_1−*x*_/CdTe absorber (≈3 µm in current devices; 2–8 µm across generations), ZnTe back contact, rear conductor and encapsulant	Thin (∼0.3–0.7 µm) halide perovskite absorber with charge selective layers and metallic electrodes (often Au or Ag)
Key features affecting degradation	Si rigidity gives the absorber inherent stability. Dominant failure modes originate from encapsulant hydrolysis, metallisation corrosion, and delamination. Intrinsic mechanisms (B–O LID, oxygen precipitation, LeTID) account for a small fraction of field failures. (Fig. 4)	Degradation driven by grain boundary instability, Cu diffusion from back contacts, and interface delamination. (Fig. 5)	Sensitive to moisture, oxygen, heat, and light. Prone to ion migration, phase segregation (*e.g.* I/Br), interface instability with transport layers, and metal diffusion. (Fig. 6)
Absorber material intensity (t GWp^−1^) (assumptions: Table S1)^57–61^	c-Si 1499 (PERC)/1377 (TOPCon); dopants ≪1	Te ≈ 48; Cd ≈ 43	Pb 3.6; I 5.4; Br 0.70; Cs 0.11 (MA/FA synthetic, ≪1)
Production yield (%)	≥95 (ref. 62)	90–96 (ref. 63)	<10 (spin coating)^64^
			≈99 (slot die)^65^

a Abbreviations: BSF, aluminium back-surface field (legacy Si-PV cell technology); B–O LID, boron–oxygen light-induced degradation; EVA, ethylene-vinyl acetate encapsulant; LeTID, light- and elevated-temperature-induced degradation; PERC, passivated emitter and rear cell (incumbent Si-PV cell technology); R2R, roll-to-roll processing; TCO, transparent conducting oxide; TOPCon, tunnel-oxide passivated contact (incumbent Si-PV cell technology).

It is important to distinguish absorber material requirements from those of the complete photovoltaic device. The absorber-focused comparisons made here are intended to isolate the consequences of absorber chemistry and should not be interpreted as a complete measure of device-level material intensity, manufacturing complexity or cost. Contact and selective/transport layers, transparent conducting oxides, metallic or carbon electrodes, substrates and encapsulation are all required to produce functioning devices and can contribute substantially to both material demand and processing complexity. Their relative importance is also strongly architecture-dependent: crystalline-silicon technologies employ different contacting and passivation schemes across PERC, TOPCon and SHJ devices, while perovskite photovoltaics currently encompass a particularly diverse range of transport layers, electrodes and device architectures.

## 3 Manufacturing

### 3.1 Silicon manufacturing

Si production begins with the carbothermic reduction of quartz (SiO_2_) in submerged-arc furnaces at approximately 2000 °C to yield metallurgical-grade silicon (MG-Si, ∼98–99% Si). This step alone requires about 11–13 MWh per t Si and constitutes the main source of early-stage emissions due to high electricity use and CO/CO_2_ off-gas generation.^66,67^ Because residual impurities and carbon–oxygen complexes in MG-Si severely limit minority-carrier lifetimes, silicon must be purified to 6–9 N purity through the Siemens process, in which metallurgical-grade silicon is first converted to trichlorosilane gas, which is then decomposed by chemical vapour deposition to yield high-purity polysilicon. This process is highly energy-intensive: overall energy consumption has historically ranged from 90–140 kWh kg^−1^ in large-capacity plants, falling to around 70 kWh kg^−1^ in the most recent generation of facilities, with the CVD deposition step alone accounting for 45–50 kWh kg^−1^ in the best case.^68^ Purified polysilicon is subsequently re-melted for crystal growth. The Czochralski (Cz) process, now dominant for high-efficiency Passivated Emitter and Rear Cell (PERC), Tunnel Oxide Passivated Contact (TOPCon), and Single Heterojunction (SHJ) architectures, requires approximately 30 kWh kg^−1^ under industrial conditions.^69^

Maintaining crystal perfection through these high-temperature processes is essential, as structural defects formed during crystal growth reduce minority-carrier lifetime. Subsequent cell fabrication steps, including phosphorus diffusion gettering and hydrogen passivation, further reduce bulk defect density before the junction is formed.^70^ The wafering stage slices the grown ingot into thin substrates and remains a notable source of material and energy loss. During cutting, the wire saw removes a thin layer of silicon, known as kerf, which is lost as fine particles and cannot be recovered into usable wafers. Although kerf loss has been reduced through thinner wires and improved slurry recovery, the material removed during cutting still represents both a yield and embodied-energy penalty, since the silicon lost had already undergone the full energy-intensive purification and crystal growth process.^71^

Overall, most of the manufacturing energy in c-Si PV arises from polysilicon refinement, ingot crystallisation, and wafering, reflecting the high-temperature and energy-intensive nature of these unit operations. Electricity provides roughly 80% of total manufacturing energy input, with polysilicon and ingot production together accounting for about two-thirds of total electricity consumption owing to their long, high-temperature heating requirements. Recent LCAs for crystalline-Si PV (U.S. utility-scale baseline) attribute the bulk of cradle-to-gate energy use and greenhouse-gas emissions to these three stages, with relative contributions modulated by technological level and grid carbon intensity. The grid mix strongly influences life-cycle footprints: coal provides ∼62% of the electricity used for solar-PV manufacturing, well above its 36% share in global generation, because most production is concentrated in China, where coal often supplies more than 75% of the power.^72^ Nevertheless, the operational energy payback of c-Si PV remains short. 1 GW of installed PV capacity can offset about 1.5 Mt CO_2_ per year from coal-fired generation, and modules typically require only 3–5 months of operation to repay their manufacturing-related emissions (a manufacturing-emissions payback, distinct from the energy payback times in Table 2). Even when full lifecycle components are included, payback times remain well below system lifetimes of 25–30 years.^72^

**Table 2:** Economic and energy metrics of c-Si, CdTe and halide-perovskite PV, with demonstrated and assumed lifetimes and the evidence level of each value^a^

	Absorber material		
	Silicon	CdTe	Perovskites
CAPEX ($ per W)	0.20–0.30 (ref. 74)	0.3–0.7 (ref. 75 and 76)	0.15–0.21 (module, triple mesoscopic)^77^
			0.04–0.1 (film, R2R)^78^
LCOE (¢ per kWh)	2–8 (ref. 79 and 80)	4–7 (ref. 79)	1.5–25.5 (ref. 77, 79, 81 and 82)
Energy payback—system NREPBT (years)	1.2 (mono-Si BSF)	0.9 (ref. 83)	No commercial system exists
Energy payback—module-only EPBT (years)	0.65 (2020 PERC)^b^	0.19 (Series 7)^b^	0.19–1.1^c^ (landfill EoL)
	2.4^c^	0.63^c^	0.09–0.93 (with recycling)
Embodied energy of modules (MJ m^−2^)	2000	530 (ref. 84)	No manufacturing-scale data; the modelled EPBT above serves as proxy
Embodied carbon of modules (kg CO_2_ eq per m^2^)	160	43	4.59 (ref. 85) (calculated from Zhao *et al.*)
Warranty lifetime (years)	25–30 years product/performance industry-standard warranty^58^	30 years (0.3% per year covered by warranty; ≈92% at year 30 (ref. 86))	None at module scale; no bankable warranty yet^87^
Field degradation rate (% per year)	0.5 (module-level, literature median)^88^	≤0.3–0.5; 0.3 covered by warranty^86^	None demonstrated; best outdoor: 78% after 1 year (mini-modules);^90^ −2.8% over 3 years (sub-modules)^91^
	0.75 (system-level performance loss, 7.2 GW US fleet)^89^		
Lifetime assumed in LCOE/EPBT/LCA (years)	25–30 (LCA convention)^57^	30 (ref. 84)	15–25, modelled only—15 years used as a stated ‘conservative’ assumption^92^
Evidence level	Field-proven; >30 years fleet statistics	Commercial, field-proven (GW scale, multi-decade)	Laboratory/pilot; outdoor data ≤3 years and small-area; EPBT and embodied-carbon values are modelled

a CAPEX, capital expenditure of manufacturing capacity per watt of annual output ($ per W). LCOE, levelised cost of electricity (¢ per kWh); depends on each source's assumed lifetime and discount rate. NREPBT, non-renewable energy payback time (IEA PVPS Task 12): values refer to a complete 3 kWp rooftop system in Europe (975 kWh per kWp per year, 30-year panel service life^83^) and scale inversely with irradiation. Module-only EPBT is computed on total cumulative energy demand (CED); the two energy bases differ by only a few per cent for PV manufacture. Embodied energy: cradle-to-gate primary energy of module manufacture per m^2^—total CED for c-Si;^93^ total primary energy (A1–A3) per the manufacturer EPD for CdTe.^84^ Embodied carbon: cradle-to-gate GWP_100_ per m^2^ of module. EPBT and the per-m^2^ embodied metrics are lifetime-independent; lifetime enters only the LCOE.

b Derived from the embodied-energy row at the module-only basis (Southern Europe, 1700 kWh per m^2^ per year, PR 0.75, grid efficiency 0.30; Section S3).

c From the same-basis comparison of Tian *et al.*;^92^ its c-Si and CdTe inventories predate 2020 manufacturing improvements.

From an economic standpoint, capital expenditure (CAPEX) is similarly concentrated in the high-temperature and high-purity segments. Equipment for Siemens CVD, Czochralski crystal pulling, and trichlorosilane refining together account for roughly 45% of total factory CAPEX. For a competitive 2 GW_a(c_ap_)_ integrated polysilicon-to-module facility, total investment is approximately US $2 billion; a plant purchasing external polysilicon requires about US $1.4 billion. These upstream investments alone represent ≈22% of module minimum sustainable price (MSP). Halving CAPEX could reduce MSP by ∼15%, illustrating how manufacturing scale and capital efficiency directly affect module costs and industry scalability.^73^

### 3.2 CdTe manufacturing

CdTe thin-film solar cells (Fig. 2) are deposited in a superstrate configuration, meaning that the active layer is deposited on top of the front glass and transparent conductive oxide (TCO).^94^ Glass represents ≈80–85% of the module weight and ≈47–54% of the module material value.^72,95^

**Fig. 2:**
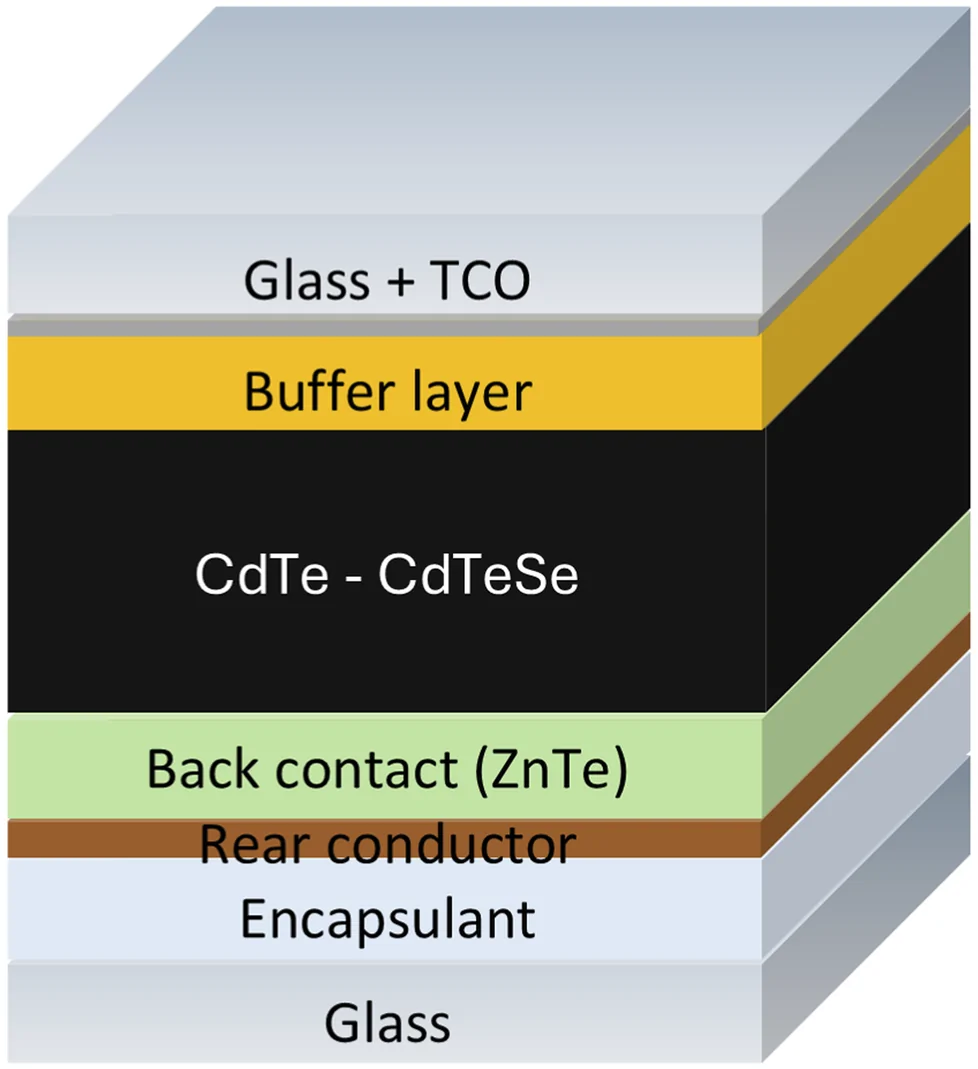
Representative layer structure of a superstrate CdTe solar cell, showing the principal functional layers from the glass substrate to the rear contact.

#### 3.2.1 Active layer (CdTe–CdTeSe)

The active layer is a p-type thin film whose composition has evolved from CdTe into an alloy of CdSe_1−*x*_Te_*x*_ and is commonly deposited *via* physical vapour deposition.^24^ This layer represents only about ≈0.4% of embodied energy (≈3 MJ m^−2^)^95^ with Te representing more than 3% of the total cost of the module (3–6%) despite its low weight percentage (0.05–0.07%).^72^ After deposition, the active layer is typically exposed to CdCl_2_*via* sublimation, evaporation or immersion, followed by annealing at 400 °C.^24^ This treatment promotes grain growth, recrystallisation, and passivation of grain boundaries and imperfections, improving the efficiency of the module.^24^

#### 3.2.2 Back contact (ZnTe), rear conductor and encapsulation

After the active layer, a p-type back contact is deposited to form an ohmic contact. This is achieved by sputtering a p-type ZnTe layer.^24^ The modules then go through laser etching and the deposition of an insulator to separate individual fingers/cells in the module, followed by a second laser etching that will create a pathway for interconnection when the rear conductor is sputtered on the back of the module. After the sputtering of the rear conductor, a third laser etching is employed to isolate the back of the cells. Finally, the individual cells are interconnected, and the module is encapsulated with EVA and the use of back glass, steel or polymers.^24,96^

Reported embodied-energy values for CdTe modules range from 76.5 to 1971 MJ m^−2^.^95,97,98^ This wide variation largely reflects differences in manufacturing vintage, system boundaries, electricity mix and the inclusion of frames and other balance-of-module components, rather than intrinsic differences among CdTe products. Using the cradle-to-gate inventory of Wikoff *et al.*, which evaluates CdTe and silicon on a consistent basis, CdTe modules embody 794 MJ m^−2^, compared with 2297 MJ m^−2^ for silicon modules.^95^ Within the CdTe inventory, module assembly contributes approximately 33% of embodied energy, the aluminium frame 32% and the glass 30%, whereas the absorber contributes only around 0.4%. By contrast, silicon wafers account for approximately 76% of the embodied energy of a silicon module.^95^ Thus, the semiconductor defining the technology contributes negligibly to the embodied-energy burden of CdTe modules but dominates that of silicon modules.

CdTe accounts for approximately 5% of global module production.^72^ Manufacture is dominated by a single vertically integrated producer, First Solar, which deposits every layer of the device in one continuous process. Integration supports reproducibility, in-process quality control and product traceability, and allows new plants to be built and commissioned in as little as 18 months.^98^ A recent 3.5 GW line with integrated recycling represented an investment of approximately US $1.1 billion, or ≈US $0.3 per W of annual capacity, placing it between the c-Si and perovskite values in Table 2.^76,98^

### 3.3 Perovskite manufacturing

Unlike crystalline silicon and CdTe, halide perovskite solar cells have not yet reached commercial module production at scale, and the manufacturing and lifecycle data discussed here are therefore based on laboratory-scale devices and modelling studies. While these projections are broadly consistent across the literature, they carry greater uncertainty than the mature datasets available for silicon and CdTe, and actual values will depend on the specific architectures, encapsulants, and processing routes adopted at industrial scale.

Despite this, projections consistently point to significant manufacturing advantages over silicon. Lead halide perovskites can be deposited from solution or vapour at low temperatures, typically below 150 °C. Their defect tolerance and soft ionic-covalent bonding permit high optoelectronic quality even when processed by scalable coating or printing methods such as slot-die, blade, gravure, or roll-to-roll deposition. Industrially relevant line speeds and film uniformity have already been achieved under modest thermal budgets.^99^ These attributes enable short, low-temperature process sequences that rely on compact tooling and modest energy input.^100,101^

Perovskite photovoltaics cut embodied energy by ∼78% and reduce GHG emissions to ∼10.7 g CO_2_ eq per kWh—around 60% lower than silicon because they eliminate the high-temperature purification and crystallisation steps required for c-Si wafers (Table 2).^102^

Energy-payback times show a similar trend. On a consistent system-level basis, all-perovskite tandems are modelled at 0.35 years, compared with 1.44 years for perovskite–silicon tandems and 1.52 years for silicon.^102^ These values are not directly comparable with the module-only estimates in Table 2, which use different system boundaries and irradiation assumptions. The projected advantage arises primarily from low-temperature absorber deposition and avoidance of silicon purification and crystallisation.^102^ Earlier studies also show that perovskite modules achieve the shortest energy-payback times among thin-film technologies^103^ and consistently lower embodied carbon across diverse manufacturing scenarios, establishing them as among the least energy- and carbon-intensive PV materials under development.^104^

At the factory level, the low-temperature, solution-compatible processing of perovskites also translates into moderate capital requirements. Economic modelling by Mathews *et al.* indicates that the influence of equipment capital on the minimum sustainable price diminishes rapidly with throughput, as material and utility costs dominate at moderate scales.^105^

Solution processing itself introduces reproducibility and yield constraints. Film formation is highly sensitive to precursor stoichiometry, solvent purity and ambient conditions, so small variations can alter phase and morphology.^106^ Achieving industrial uniformity thus requires controlled environments and *in situ* diagnostics, offsetting some of the simplicity of the chemistry. Assessing manufacturing viability requires that technology readiness be evaluated on consistent terms. One approach is to consider only scalable methods and materials, correlating them with the spread of performance achievable under those conditions; this also provides a baseline for optimisation. Alternatively, laboratory-scale performance may be reported, but it must be paired with the penalties such methods impose on economic viability and process simplicity. Presenting efficiencies achieved by methods with no route to large-area deposition alongside projections for industrial scale risks overestimating technology readiness.

The practical advantages of low-temperature, solution-based processing are counterbalanced by challenges rooted in the chemistry of halide perovskites. The most immediate limitations are reproducibility, stability, and the toxicity of lead. Encapsulation layers must therefore provide exceptional moisture and oxygen barriers, besides chemical and thermal compatibility with the perovskite layer^107^ and lead containment during breakage;^108^ their production can increase the module's embodied energy.^81^ Additionally, the encapsulant material should facilitate disassembly/delamination and recovery and reuse of materials at the EoL, a challenge currently shared by all PV technologies.^109^

Finally, economic maturity lags behind established technologies. Supply chains for perovskite precursors, electrodes and encapsulants are still fragmented, limiting scale economies and cost predictability.^81^ Moreover, most life-cycle assessments assume multi-decadal lifetimes that have yet to be proven in field operation. Shorter operational lifetimes would narrow the current energy and carbon advantage.

### 3.4 Supply risks

Supply risk reflects vulnerabilities of PV technologies to limited raw material availability, arising from geological scarcity, geographic concentration, by-product dependency, and recycling limitations (Table 3). Fig. 3 maps the supply-risk index (SRI, 0–10) of elements used in the absorber and directly associated layers of the three PV technologies, calculated from six weighted indicators, namely: geological scarcity, production concentration, by-product dependence, recycling deficit, PV demand pressure and refining concentration, with global scope and 2024/25 reference data (methodology, inputs and sensitivity in SI S2, Tables S2–S6). Component profiles of the five elements classed as high or above (SRI ≥ 7) are shown in Fig. 3b. Traditional c-Si absorber materials, including Si as the absorber and P and B dopants, are geologically abundant, though high energy purification of Si is geographically concentrated with high CAPEX as a barrier to establishing new Si refining globally.^110^ Quartz sand is one of the most abundant raw materials on Earth, but environmental and governance issues in sand mining and energy-intensive refinement to 7 N Si remain challenges for sustainability rather than availability.^111,112^ P and B also exhibit some geographic concentration of reserves.^113,114^ Recent advances in high-value recycling processes for Si waste also demonstrate improved removal of boron and oxygen impurities, potentially increasing secondary supply to mitigate supply vulnerabilities.^115^

**Table 3:** Supply-risk classification, hazards and end of life characteristics of c-Si, CdTe and halide-perovskite PV^a^

	Absorber material		
	Silicon	CdTe	Perovskites
Component supply-risk class (Fig. 3, SI S2)	• High: Ga	• Very high: Te	• High: Cs
	• Moderate: Si	• High: As, Se	• Moderate: I
	• Low: B, P	• Moderate: Cd, Mg	• Low: Sn, Br, Pb
		• Low: Zn	• Very low: MA, FA, organic transport materials
		• Very low: Cu	
Toxicity/hazards related to active layer	Lead (soldering), toxic fumes generated during pyrolysis of EVA	CdTe presents acute toxicity and is very toxic to aquatic life	Problematic solvents *e.g.* DMF and chlorobenzene and lead salts during fabrication – potential toxic emission in fabrication
EoL hazards	HF, HNO_3_ used during processing	• Toxic Te compound formation during improper handling	• Fumes generated during pyrolysis of encapsulant
		• Acid fumes from EoL	• Leaking of lead ions during regeneration of substrates
		• Emissions of doping metals in leachates (As, Cu, Mg, Se, Zn)	• Use of toxic solvents during cell recycling and remanufacturing
Recycling rates (%)	• Glass 98%	• 90% (recovery of semiconductor material for reuse in new modules)	Lab-scale demonstrations report >95% Pb recovery and solvent-based substrate remanufacturing without performance loss, closed-loop approaches look promising for scale up^126,127^
	• Al 99%	• 90% (recovery of glass for use in other glass products)^98^	
	• Si 95%		
	• Cu 99%		
	• Ag 94% (ref. 124)		
	Mostly open-loop; bulk recovery dominates rarely delivering Si/Ag at grades for use in new PV^125^		

a Supply risk class: study-specific comparative indicator defined in Section 3.4 and SI S2); classes: very low ≤ 2, low > 2–4, moderate > 4–6, high > 6–8, very high > 8–10. Values are comparative indicators, not official EU CRM supply-risk scores. CRM, critical raw material; EoL, end of life.

**Fig. 3:**
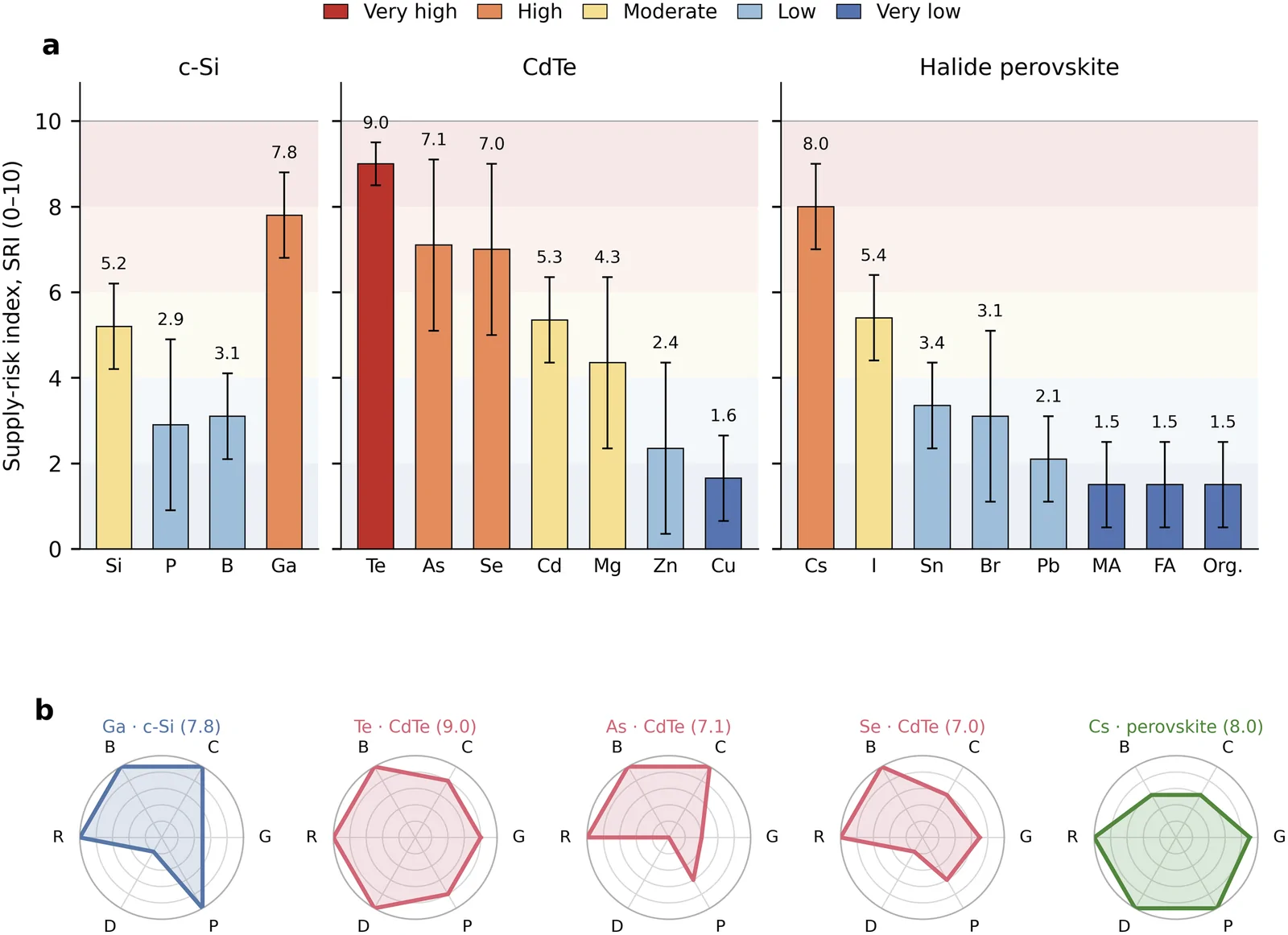
Supply risk of elements used in c-Si, CdTe and halide-perovskite PV. (a) Supply-risk index (SRI, 0–10), a weighted composite of six indicators (global scope, 2024/25 data; methodology, sources and sensitivity in SI S2). Error bars show data-quality bands; shading indicates the five risk classes. Values are study-specific indicators. (b) Six-indicator profiles of elements with SRI ≥ 7 (G: geopolitical scarcity, C: production concentration, B: by-product dependence, R: recycling deficit, D: PV demand pressure, P: refining concentration).

Despite this low susceptibility to supply risk, high-efficiency c-Si PV increasingly uses Ga in place of B as the p-type dopant, eliminating B–O light-induced degradation (B–O LID), while n-type architectures rely on P; As is not used in mainstream c-Si manufacturing, though it is an emerging dopant in CdSeTe absorbers. The shift to Ga, however, introduces greater supply risk. Ga is not directly mined but recovered as a by-product of bauxite and zinc refining, with China accounting for ≈98% of low-purity output. As, which is relevant as a CdSeTe dopant, is similarly obtained as a by-product of Cu, Au and Pb ores, with Peru the leading producer and the top three producing countries accounting for >95% of global supply. By-product supply cannot easily scale in response to demand spikes, and production is geographically concentrated, placing both elements in the high supply-risk class (Fig. 3).^116^

In CdTe PV, supply risk concerns are dominated by Te, which is extremely scarce and >90% produced as a by-product from anode slimes in copper refining, giving it the highest supply risk of all PV absorber elements.^60^ Availability of Te may limit the future market share of CdTe, and secondary supply from EoL modules is limited at this time owing to long product lifetimes and low volumes of modules reaching EoL.

Cd is more stable, but its production remains linked to Zn markets and mining volumes.^116^ Long-term dynamic criticality analyses suggest that Te remains the most vulnerable, while Se, which is alloyed in CdTe, is classed as high owing to by-product dependence and increasingly concentrated refining (China accounts for ≈53% of refined output). Cd faces lower supply risks in the future.^117^ Despite extreme supply risk, long-term field data show that CdTe modules exhibit superior durability and lower degradation than other thin-film options, improving their attractiveness and sustaining pressure on tellurium supply.^118^ Broader PV deployment scenarios to 2050 identify Ga, As, and Se as consistently high-risk elements, reinforcing their importance across multiple technologies.^119^ Alloying and dopant elements such as Cu, Zn, and Mg carry lower risks: Cu is abundant (with high recycling rates),^110^ Zn is widely mined^113^ and Mg is abundant, resulting in very low risk.^110^ Sustainability assessments indicate that although Cd and Te raise toxicity concerns, industrial practice and EoL recycling ensure minimal environmental release and high recovery potential, further emphasising that their main challenge is one of supply rather than environmental safety.^120^ The only realistic mitigation strategy for Te supply is increased recycling, while Cd management is feasible under controlled industrial conditions.^7^

For perovskite PV, the picture is mixed. Pb is abundant and readily recycled, but Sn (the main candidate as a substitute for toxic Pb in the perovskite absorber) is often cited as higher-risk owing to geographically concentrated and politically unstable supply chains, although, based on current production and recycling data, it is classed as low (Fig. 3), with its risk increasing if Sn-based absorbers reach scale.^116^ Cs is of very high concern, as it is sourced from a handful of deposits worldwide.^59,121^ In contrast, alternative organic cations such as methylammonium (MA^+^) and formamidinium (FA^+^) are fully synthetic and carry negligible risk, as their supply can be scaled with demand.^59^ Among the halides used in perovskite structures, Br is of low supply risk, as it is sourced from brine deposits in the USA, Israel, Jordan and China, whereas I is of moderate risk: more geographically concentrated (Chile ≈65% of production), subject to competing industrial demand, and with projected perovskite demand reaching ≈37% of current production at 1 TWp per year deployment.^122^ Large-scale deployment modelling of perovskite tandems further underlines that Cs and Sn pose the highest long-term supply risks, while halides are of only moderate concern, confirming that mitigation strategies should prioritise scarce inorganic elements rather than synthetic organics.^59^ EU level assessment reinforces that overall for PV materials Sn, Ga and Te represent acute supply risks for solar PV in Europe, while Cu and Se remain of medium concern, linking supply chain vulnerabilities directly to strategic autonomy debates.^123^

### 3.5 Centralised *vs.* distributed manufacturing

Silicon's upstream purification, ingot growth and wafering favour large, capital-intensive and geographically concentrated plants. These operations demand far greater investment than downstream cell and module assembly, which tends to be more widely distributed in the form of assembly industries across the world, with viable plants as small as 100 MW per year and requiring only a few million USD in initial investment. According to the IEA Special Report on Solar PV Global Supply Chains (2022), the infrastructure cost of establishing a polysilicon plant typically ranges from USD 200–400 million, and facilities smaller than about 10 000 t Si per year (equivalent to roughly 3 GW per year of module output) are generally not financially viable. To achieve lower investment intensity per watt, manufacturers therefore rely on very large facilities: newly constructed polysilicon complexes in China commonly range from 40 000–100 000 t per year, almost triple historical averages, with investment intensities of approximately USD 60 million per GW. Similar costs are reported for ingot and wafer manufacturing plants operating at 5–20 GW per year, about five times the capacity of comparable facilities elsewhere. This strong dependence of cost on scale explains the high degree of industrial centralisation observed in these segments of the silicon PV industry, with China now accounting for over 80% of global PV manufacturing capacity and nearly 95% of polysilicon, ingot and wafer production.^111^ Deployment of this manufacturing capacity in Europe would require a similar pattern of large, gigawatt-scale factories that are fully integrated across the value chain, from ingot and wafer to cell and module, and highly automated to take advantage of scale efficiencies.^114^ With the advantage of green energy being more readily available in Europe to provide the required electricity, such facilities could achieve a lower carbon footprint.^111,115^ Other independent analyses support this link between factory scale, integration and cost reduction. Helveston *et al.* showed that globally scaled and integrated PV supply chains achieve module prices 20–30% lower than nationally fragmented production, while reshored or regional PV manufacturing becomes cost competitive only when operated at large, integrated scales and powered by low-carbon electricity.^128^

CdTe production is also highly centralised, but for reasons distinct from those governing silicon manufacturing. Silicon production is concentrated primarily because of its capital intensity and economies of scale, conditions that can, in principle, be replicated wherever sufficient investment and low-carbon electricity are available. CdTe production is shaped instead by the combined demands of material stewardship and constrained tellurium supply. Continuous monolithic deposition, containment of cadmium throughout manufacturing, and the recovery of tellurium from EoL modules favour a vertically integrated production and recycling model; the industry has consolidated accordingly (Section 3.2). The principal constraint on expansion, however, is not capital alone. Because tellurium is recovered predominantly as a by-product of copper refining, its supply cannot readily increase independently of copper production (Section 3.4). Large-scale investment could therefore relocate or expand silicon manufacturing, whereas increasing CdTe capacity remains fundamentally constrained by access to tellurium.

The defect-tolerant nature of the perovskite absorber allows for low-temperature, solution-compatible processing. This translates into moderate capital requirements that contrast with those of crystalline silicon. The moderate capital requirements of perovskite manufacturing, discussed in detail above, have a geographic dimension that extends beyond cost. Because viable production does not require the gigawatt-scale facilities and multi-hundred-million-dollar investments that characterise polysilicon plants, perovskite manufacturing could, in principle, be established at a regional or national scale in regions where the capital intensity of silicon excludes domestic production capacity and limited energy access.^129^ This remains a prospective advantage rather than a demonstrated industrial outcome. Controlled environments, solvent recovery, precision coating, and the contribution of encapsulation, quality assurance and certification could still create substantial barriers.

## 4 Degradation mechanisms

The chemical rigidity and insolubility of crystalline silicon and CdTe that underpin their stringent manufacturing requirements also prove advantageous during operation. Silicon's strong covalent bonding and CdTe's stable ionic-covalent lattice resist chemical degradation under field conditions, underpinning the long operational lifetimes characteristic of both technologies. Crystalline silicon modules exhibit degradation rates of only about 0.5–0.6% per year, supporting stable operation well beyond 25 years.^130,131^ This stands in contrast to halide perovskites, where the soft ionic lattice and mixed organic–inorganic bonding that enable low-temperature processing also render the material inherently more susceptible to degradation.

### 4.1 Silicon PV degradation mechanisms

Durability does not mean immunity to degradation. While silicon's chemical rigidity underpins its long operational lifetime, certain residual impurities and defect complexes, particularly those introduced during Czochralski growth, can progressively limit carrier lifetime if not adequately mitigated during manufacturing or subsequent passivation. The following are intrinsic mechanisms of degradation found in crystalline silicon.

#### 4.1.1 Boron–oxygen light-induced degradation (B–O LID)

During Czochralski (Cz) growth, oxygen from the quartz crucible (99.997% SiO_2_) dissolves into the molten silicon at approximately 1400 °C and becomes incorporated into the crystal as interstitial oxygen (O_i_).^20,132^ Under illumination of B-doped Cz-Si, interstitial oxygen and boron form a metastable B–O complex that acts as a deep recombination centre. This complex is activated under carrier injection and deactivated (or transformed) through specific thermal/illumination regeneration treatments. Passivation of this complex is achieved *via* hydrogen-assisted pathways that deactivate the recombination centre.^132,133^ In practice, B–O LID manifests as an early-life efficiency drop dominated by loss of minority carrier-lifetime (V_OC_, sometimes FF), followed by partial or near-complete recovery if passivation is successfully applied. Without passivation, cells stabilise at a lower minority carrier-lifetime plateau.^132,134^

#### 4.1.2 Light- and elevated-temperature-induced degradation (LeTID/LETID)

LeTID is a bulk defect phenomenon that appears after exposing finished cells/modules to elevated temperature (60–120 °C) under illumination. Hydrogen introduced during firing/passivation for the B–O LID recombination interacts with pre-existing defects to form lifetime-killing complexes that are injection-activated. The resulting power loss, primarily in V_OC_ and FF, develops over weeks to months but is partially reversible under continued light and heat stress, leading to loss-recovery behaviour whose long-term balance depends on climate and operating temperature.^135–137^

#### 4.1.3 Oxygen-related defects in Cz-Si

During cooling and later thermal steps, interstitial oxygen from the quartz crucible can precipitate and cause associated dislocations/stacking faults. The precipitates and their strain fields introduce Shockley–Read–Hall recombination centres, reducing minority-carrier lifetime and sometimes altering resistivity; their density and electronic activity depend on temperature history and dopant type.^138^ In PV-grade Cz-Si, oxygen-precipitation-related defects therefore impose a baseline recombination penalty that can limit stabilised efficiency, and, because precipitates can evolve with thermal exposure, also influence long-term stability.^20^

#### 4.1.4 Potential-induced degradation (PID)

Under high system bias to ground (hundreds of volts), alkali ions, mainly Na^+^ from soda-lime glass, can migrate through the encapsulation to the cell, while the electric field also changes the fixed charge at interfaces. Depending on technology and bias polarity, PID can manifest as shunting (PID-s), polarisation/charge effects at dielectrics (PID-p), or corrosion-assisted losses, each leading to rapid power loss through decreased shunt resistance, reduced lifetime, and/or FF collapse.^139^

The mechanisms described above represent performance loss pathways intrinsic to or directly involving the silicon absorber, yet empirical field data suggest that these account for only a small fraction of actual module failures. LID, the most directly absorber-related failure mode, is observed in only 0.5–5% of cases of early failure and is only classified as a failure when the saturated power loss surpasses the expected limits already accounted for in the module's rated power specification. The dominant failure modes across all lifetime phases originate instead in optical degradation, junction box and cable faults, delamination, glass breakage, encapsulant discolouration, and interconnect corrosion, rather than in the silicon absorber itself. The mechanisms more likely to cause decommissioning are therefore those discussed below. A summary of all mentioned mechanisms of degradation is shown in Fig. 4.

**Fig. 4:**
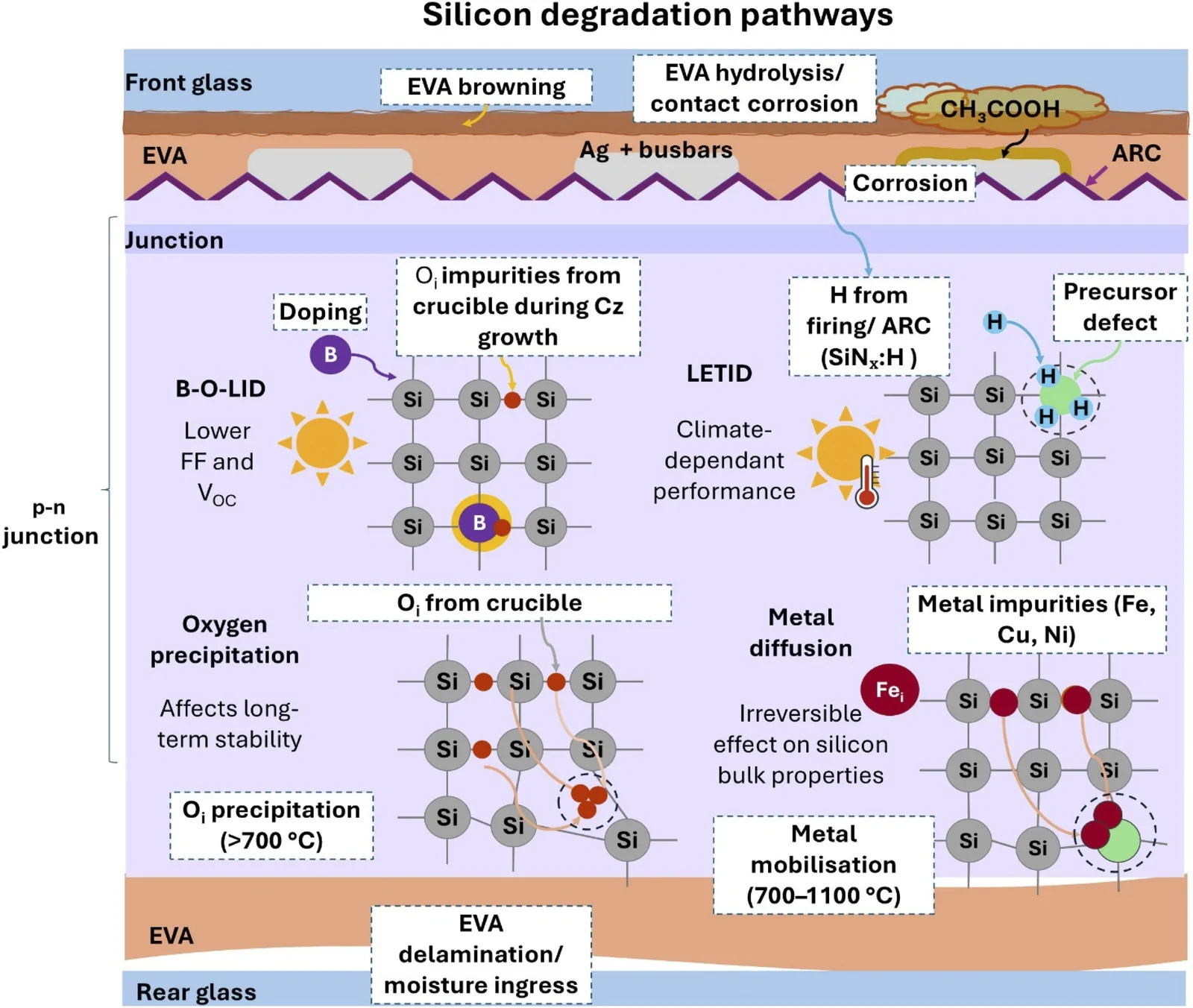
Schematic overview of intrinsic silicon-wafer degradation mechanisms (boron–oxygen light-induced degradation (B–O LID), light- and elevated-temperature-induced degradation (LeTID), oxygen precipitation, and metal diffusion) and module-level degradation pathways (EVA browning, EVA hydrolysis, delamination, moisture ingress, and corrosion). These processes generate deep electronic traps or shunting paths that reduce carrier lifetime, V_oc_, FF, and long-term module stability.

#### 4.1.5 Metallisation and interconnect corrosion driven by EVA hydrolysis (acetic acid formation)

Ethylene-vinyl-acetate (EVA) encapsulants can degrade under heat, moisture and UV exposure to produce acetic acid, which can accumulate within modules to promote corrosion of cell metallisation, solder bonds and Cu-based interconnects. The resulting gridline and ribbon corrosion increases series resistance and can ultimately lead to interconnect failure, a degradation pathway that is particularly important in hot and humid climates.^140–142^

#### 4.1.6 Delamination and moisture ingress

Loss of laminate adhesion (glass/EVA(or POE)/cell/backsheet) and micro-pathways at edges or interfaces enable moisture ingress that accelerates multiple electrochemical and interfacial failure chains. Water and oxygen permeating the stack hydrolyse EVA, generating acetic acid that corrodes Ag/Cu/Al metallisation and busbars, increases series resistance, and promotes local shunts and hot-spots; loss of adhesion further compromises the barrier, creating positive feedback between ingress and corrosion^140,143,144^

#### 4.1.7 Optical, thermal and mechanical degradation

Optical degradation originates from soiling, encapsulant yellowing, and coating abrasion lowering transmittance and increasing localised heating.^145^ Thermal stress and partial shading create hot spots that accelerate metallisation corrosion, solder creep, and polymer ageing,^140,146^ while cyclic thermomechanical loads induce solder-bond fatigue and interconnect corrosion, raising series resistance and causing open circuits over time.^147^ Mechanical stress, including micro-cracking from transport or snow and wind loads, progressively isolates active cell areas and exacerbates mismatch and heating.^148^ Back-sheet embrittlement or cracking further opens moisture pathways that amplify corrosion and insulation faults.^144,149^ Together, these mechanisms interact across optical, electrical, and mechanical domains, determining the durability of PV modules under real-world operating conditions.

### 4.2 CdTe PV degradation mechanisms

For commercial CdTe devices, most of the degradation is related to the encapsulant material, the electrodes and the nature of its superstrate configuration, where the active layer is deposited on glass, making it particularly susceptible to breakage.^94^ The estimated degradation rates for modules are reported between 0.2 and 4% per year with a median value of 0.5% per year,^94^ while First Solar “Series 7” is covered by a warranty for an annual degradation rate of 0.3%.^86^ However, the active layer can still interact with elements of the device or the environment, leading to degradation (Fig. 5).

**Fig. 5:**
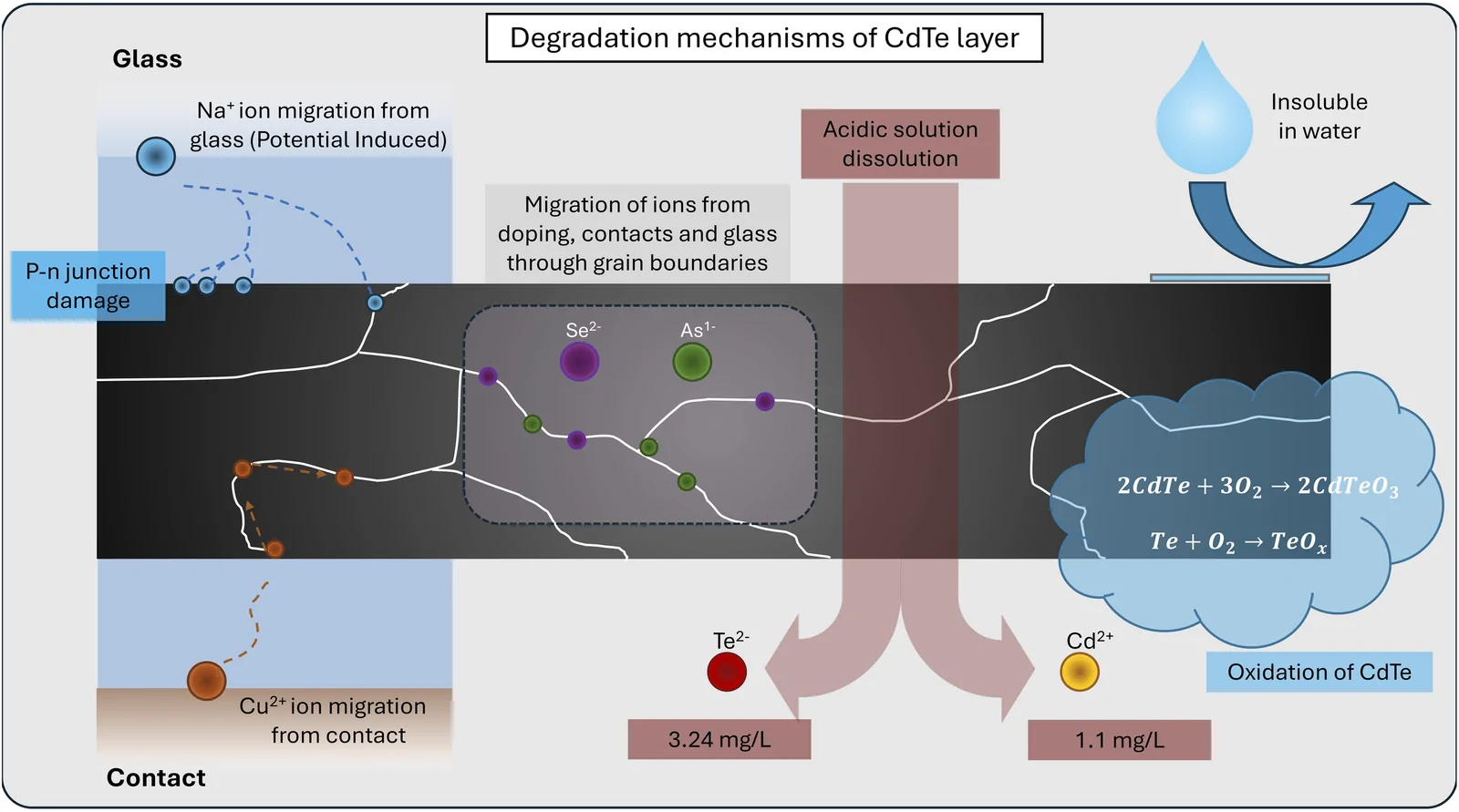
Principal degradation pathways in CdTe solar cells, showing the effects of moisture ingress, ion migration, interfacial reactions and contact degradation on device performance.

#### 4.2.1 Interaction of CdTe with water

CdTe is insoluble in water. Water-related degradation mechanisms in a CdTe device are then related to the oxidation of the back contact and the peeling of the front contact.^150^ However, in an accelerated column test, Ramos-Ruiz *et al.* showed that exposed CdTe might be susceptible to degradation in acidic solutions, (pH ≈ 4.7) leading to the release of Cd and Te within a timescale of 10–30 days. As highlighted by their work, this is particularly important in cases where waste from CdTe devices ends up in landfills, where acidic conditions might occur.^151^

#### 4.2.2 Interaction of CdTe with oxygen

It has been shown for CdTe nanocrystals that oxygen can react with exposed Te ions to form TeO_*x*_ species.^152,153^ Oxygen can come from oxidation of back contacts and migrate to the active layer, increasing the series resistance of the device.^154^ CdTe can also oxidise to CdTeO_3_ at temperatures higher than 350 °C, although the kinetics for oxidation are very slow at room temperature.^155^

#### 4.2.3 Interaction with impurities

Impurities from raw materials, those released from the substrate and contacts, doping agents and ions such as Cl ions from CdCl_2_ treatment can diffuse through grain boundaries, change the doping profile of the active layer and reduce its performance. Temperature and bias can increase the diffusion of impurities.^24^

#### 4.2.4 Migration of ions from back contacts

Cu has been used to reduce resistance of the back contact for CdTe solar cells.^94^ However, Cu atoms can diffuse through Cd-deficient layers and grain boundaries of the active layer and induce degradation at the CdS layer and shunting in the device within a timescale of less than 500 hours.^24,94,156^

#### 4.2.5 Potential-induced degradation

The glass superstrate in CdTe modules is typically soda-lime glass.^94^ A large potential difference is formed between the grounded aluminium frame and the module, which can lead to migration of Na^+^ ions from the glass to the active layer, creating recombination centres, and this process can be enhanced under high humidity conditions.^94^ This process can also lead to p–n junction damage.^144^

### 4.3 Perovskite PV degradation mechanisms

Halide perovskites exhibit several forms of instability that currently limit their long-term operation. Some are intrinsic to the material and its thermodynamic and structural characteristics, while others are related to external environmental stressors.

MAPbI_3_ exhibits a form of intrinsic structural instability associated with temperature-dependent phase transitions. At room temperature, MAPbI_3_ adopts a tetragonal structure but undergoes a reversible tetragonal-to-cubic transition at approximately 330 K.^157^ This transition lies within the temperature range that PV modules can experience during outdoor operation and involves changes in octahedral tilting and lattice symmetry that can influence the electronic and photovoltaic properties of the material. At higher temperatures, thermal stability becomes an additional concern: MA-containing perovskites can degrade even in inert atmospheres, with MAPbI_3_ decomposition leading to PbI_2_ and loss of volatile MA-derived species.^158^

FA- and Cs-based perovskites offer greater thermal stability but face a different structural limitation. In the ABX_3_ structure, the size of the A-site cation influences the stability of the corner-sharing PbX_6_ framework, a geometrical relationship commonly described by the Goldschmidt tolerance factor.^159^ MA^+^ has a size that fits relatively well within the cavities formed by the PbI_6_ octahedra, allowing the three-dimensional corner-sharing framework to be accommodated with moderate octahedral distortion.^157^ In contrast, FA^+^ is comparatively large, placing strain on the surrounding Pb–I framework, whereas Cs^+^ is comparatively small, favouring greater octahedral tilting and distortion. Both therefore lie towards opposite limits of the size range favourable for the 3D iodide perovskite structure. Consequently, although FAPbI_3_ and CsPbI_3_ can form photoactive black perovskite phases, these structures are metastable at room temperature: FAPbI_3_ tends towards the yellow hexagonal δ-phase, while CsPbI_3_ tends towards the yellow, non-perovskite orthorhombic δ-phase.^160^ Persistence of the black phase therefore depends on barriers to nucleation and phase transformation. A-site alloying with cations of complementary sizes reduces structural mismatch and modifies the relative energetics of the competing phases, while lattice strain, halide alloying with bromide, and additive and interfacial engineering can adjust both the kinetic barriers and the underlying free-energy landscape.^161,162^ This stabilisation remains sensitive to composition, processing history, and external factors such as moisture and temperature. Repeated thermal cycling can promote nucleation, strain relaxation and phase evolution, while moisture can accelerate conversion to non-photoactive phases.

Beyond these structural limitations, the ionic and dynamically disordered character of the lattice renders the absorber susceptible to environmental stressors.^163^ Moisture, oxygen, operational conditions (bias and illumination) each drive distinct degradation pathways (Fig. 6).^164–166^ These stressors rarely act independently, and their combined effects can alter degradation pathways and accelerate overall material deterioration.

**Fig. 6:**
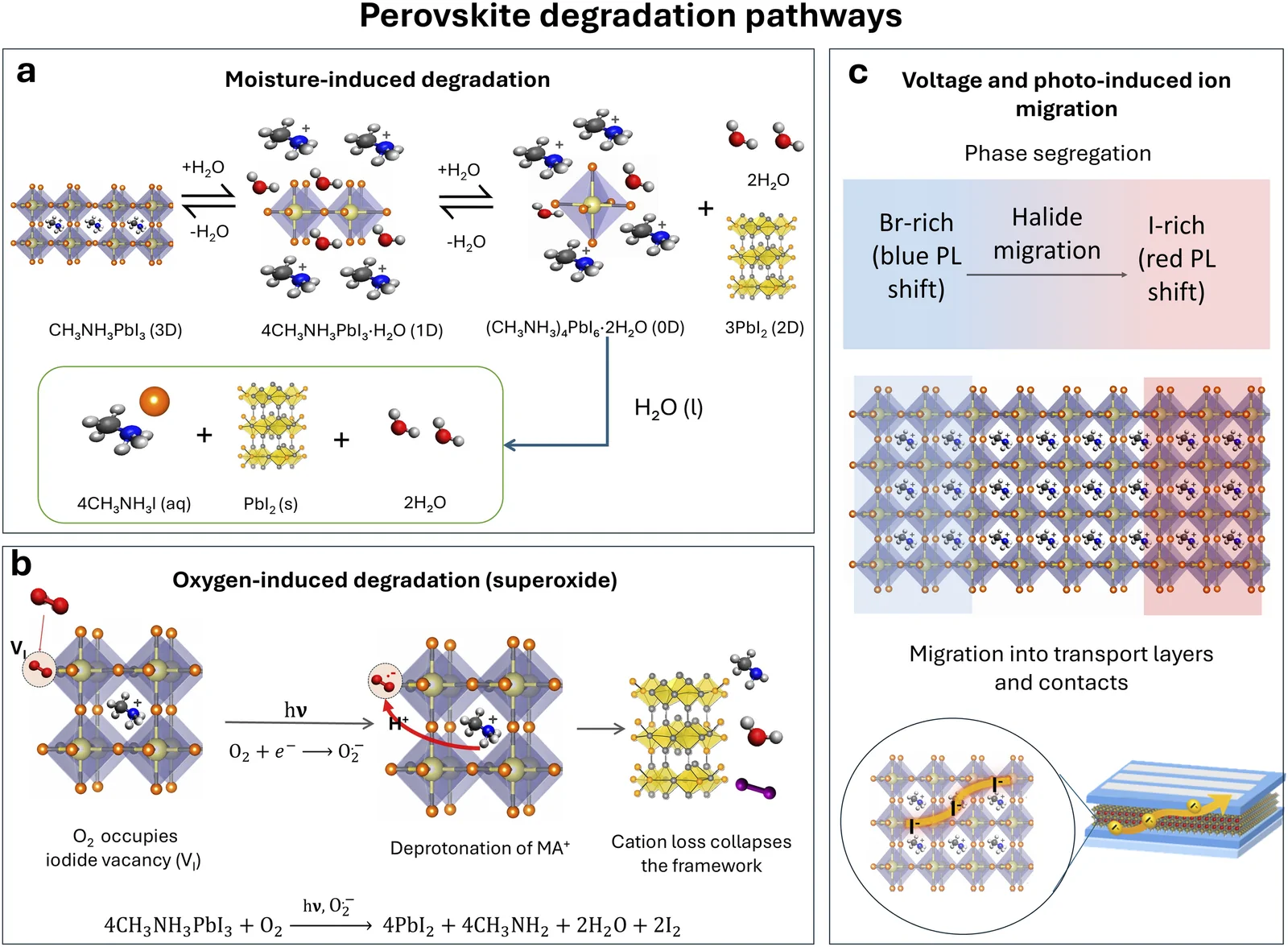
Perovskite degradation mechanisms. The relatively weak bonding in the perovskite lattice makes these materials inherently prone to degradation. These diagrams show how external factors including (a) moisture, (b) oxygen and (c) voltage and photo-induced ion migration catalyse structural changes such as phase segregation or full degradation into PbI_2_ and volatile species.

#### 4.3.1 Moisture-induced degradation

Moisture is among the most persistent stability challenges facing perovskite photovoltaics, and the susceptibility of MAPbI_3_ to humid environments is well documented. Early accounts, supported by XRD of degraded films, described a straightforward breakdown of the perovskite into HI, PbI_2_ and CH_3_NH_2_.^167,168^ Degradation under atmospheric humidity is, however, a gradual process, and time-resolved characterisation has given deeper insight into the intermediate hydrated species and reversible steps that precede irreversible breakdown of the MAPbI_3_ absorber. According to Leguy *et al.*, water molecules intercalate into the crystal structure of MAPbI_3_, inducing a rearrangement of the [PbI_6_]^4−^ octahedra from a three-dimensional (3D) network in pristine MAPbI_3_ to one-dimensional (1D) double-chains in the monohydrated MAPbI_3_ species, and finally to a zero-dimensional (0D) framework of isolated octahedra in MAPbI_3_ dihydrate. This process suggests the following reaction:^169^4(CH_3_NH_3_)PbI_3_ + 4H_2_O ⇌ 4[CH_3_NH_3_PbI_3_·H_2_O] ⇌ (CH_3_NH_3_)_4_PbI_6_·2H_2_O + 3PbI_2_ + 2H_2_O

The monohydrate can be easily converted back into MAPbI_3_ but prolonged exposure to water vapour initiates the formation of the dihydrate species and PbI_2_, with the release of two water molecules. Reversibility of the monohydrate-to-dihydrate conversion is likely limited by phase separation of the reaction products. Eventually, an excess of water may result in the dissolution of MA^+^ and the irreversible degradation of the structure:^169^
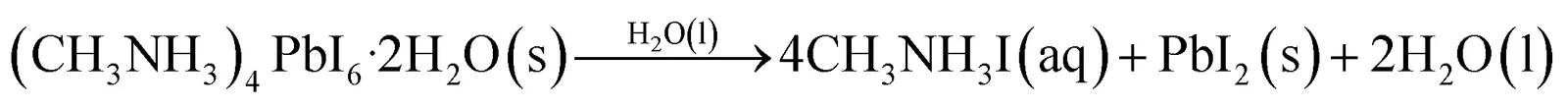


Encapsulation is the primary defence against moisture-induced degradation. Compositional engineering offers a complementary route: substitution at the A site with formamidinium, caesium or mixed cations improves resistance to humidity relative to MAPbI_3_, though it alters the degradation behaviour rather than eliminating it.^170^

#### 4.3.2 Oxygen-induced degradation

Molecular oxygen diffuses rapidly into MAPbI_3_ films, occupying iodide vacancy sites within the lattice.^165^ Under illumination, a photogenerated electron reduces the trapped oxygen to superoxide, a strong base that deprotonates the adjacent methylammonium cation. Loss of the A-site cation destabilises the framework, and the material decomposes to PbI_2_ with release of methylamine, water and iodine (Fig. 6b):
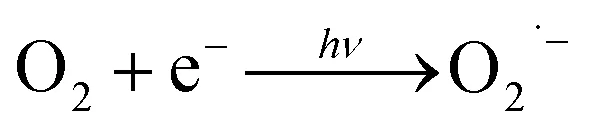

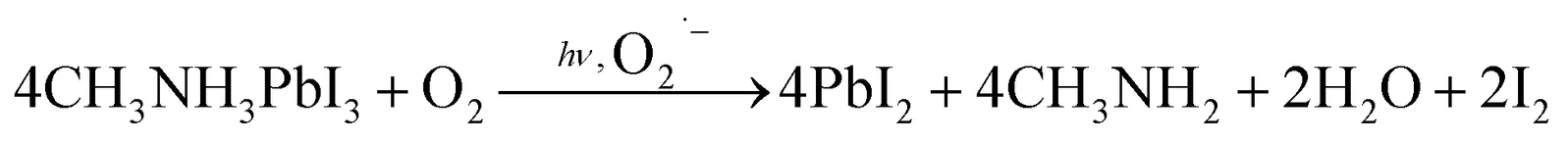


Because superoxide formation is mediated by iodide vacancies, degradation depends on defect density rather than on oxygen exposure alone: films of smaller crystallite size present more vacancy sites and degrade correspondingly faster, while passivation with iodide salts suppresses superoxide generation.^165^ Additionally, replacing the methylammonium component in MAPbI_3_ with a species without acidic protons could improve tolerance to oxygen and enhance stability. Results from Lin *et al.* suggest that the incorporation of Cs suppresses this pathway. At 5%, Cs^+^ raises the formation energy of the superoxide, while simultaneously raising the activation energy for iodide vacancy migration, restricting access to the vacancy sites where oxygen is reduced.^171^

#### 4.3.3 Light-induced degradation

Under illumination, perovskite layers are also prone to halide phase segregation, particularly in mixed-halide systems. The spatially inhomogeneous bandgap generated under light or current injection by this segregation severely limits the long-term stability of mixed-halide perovskites. Even when segregation is minimal (approx. 1%), it induces a disproportionate performance loss owing to the funnelling and concentration of charge carriers within iodide-rich regions.^54,172^

#### 4.3.4 Degradation under operational conditions and ion migration

Operational stress further accelerates degradation. Ion migration under an electric field, primarily vacancy-assisted halide migration with activation energy around 0.6 eV for I^−^ in MAPbI_3_, leads to interfacial accumulation, field-induced p–i–n redistribution, and long-term compositional drift.^173–175^ In the context of the full device degradation triggered by chemical changes originating at the active layer, halide migration also drives electrode corrosion. Iodide reaching the Ag contact forms AgI, while metal diffusion into the perovskite, including Ag diffusion again forming AgI, generates trap states and shunt paths, though gold remains comparatively inert.^176,177^ Charge-transport layers introduce additional interfacial instability. UV-excited TiO_2_ can catalyse degradation at the electron-selective contact.^178^ At the hole-selective contact, oxidatively doped spiro-OMeTAD contains radical cations that can oxidise iodide, generating iodine and additional iodide vacancies in a reinforcing degradation cycle.^179^ Hygroscopic lithium dopants further promote moisture ingress, while migration of their constituent ions under bias causes the contact composition to evolve during operation.

Combined stressors tend to amplify degradation. The interaction of light and oxygen represents the fastest degradation route for MA-based perovskites through O_2_^−^ chemistry.^165^ The synergistic presence of oxygen and moisture accelerates photooxidation and halide loss.^165^ Thermal and electrical bias together enhance ion migration and electrode reactions, compounding degradation effects.^164^ These degradation pathways ultimately limit the operational lifetime of perovskite solar cells.

Ionic movement by itself is not the sole predictor of irreversible degradation. Cells with negligible hysteresis exhibit field screening comparable to strongly hysteretic ones, suggesting that hysteresis requires mobile ionic charge and recombination near the contacts acting together.^174^ Redistribution confined to the absorber has been shown to be reversible: efficiency decreases by 10–15% over several hours at the maximum power point but recovers in the dark,^55^ and cells can lose efficiency to ionic screening of the internal field while the bulk and interfaces remain undamaged.^56^ Irreversible damage occurs when ions leave the absorber, changing its composition and undergoing further reactions with transport layers and contacts that lead to device breakdown.^176,179^ Mitigating ion-mediated degradation over operational lifetimes may therefore be a matter of raising migration barriers enough to keep redistribution reversible and contained within the absorber.

Encapsulation and composition and interface engineering have shown promising results against degradation,^180,181^ but stability characterisation of perovskite devices is not yet assessed uniformly across the field, and reported lifetimes remain difficult to compare. Stability characterisation for perovskite devices is assessed against two distinct benchmarks. The ISOS protocols are consensus procedures developed within the research community with the aim of standardising laboratory ageing tests (dark storage, light soaking, outdoor exposure, thermal cycling, and bias stress) so that stability data are comparable between laboratories; they carry no certifying force. IEC 61215, by contrast, is the industrial design-qualification and type-approval standard for terrestrial PV modules [IEC 61215-1:2021], a standardised sequence of accelerated stress tests (including damp heat, thermal cycling, humidity-freeze, UV exposure, and mechanical load) that module designs must pass with less than a defined power loss, and which is a precondition for commercial deployment, warranties, and project financing.

At the laboratory scale, a small number of studies have replicated individual IEC qualification tests on research devices. These laboratory tests replicate individual test items drawn from the standard to demonstrate that the standard's harshest environmental conditions can be met, but carry no certification (Table S7).^181–186^ Larger outdoor datasets are required to establish a realistic picture of perovskite stability that captures the distribution of performance across large batches of modules rather than the behaviour of individual champion devices. Currently, of more than 43 000 devices in the perovskite database, most have no associated stability data,^187^ and approximately 7000 reference the ISOS protocols.^188^ Moreover, outdoor degradation has been shown to depend strongly on climate. Lifetime indicators vary with season and ambient temperature,^189^ same-batch devices have degraded at a hot Mediterranean site while remaining stable at a cooler continental one,^190^ and encapsulated devices have shown partial delamination within weeks in hot climates while surviving for years in temperate ones.^191^ Stability datasets must therefore span multiple climatic zones and commercially relevant timescales to provide a realistic overview of the technology's level of stability.

## 5 End of life

### 5.1 Silicon PV EoL

#### 5.1.1 Current EoL procedures

Despite the maturity of silicon manufacturing, EoL recovery of the absorber material remains limited, with most modules entering recycling routes designed to recover glass, aluminium and metals rather than the silicon wafer itself.^192–194^Fig. 7 illustrates both the manufacturing process and the current and emerging EoL landscape, alongside alternative routes that could in principle enable silicon wafer recovery and reuse.

**Fig. 7:**
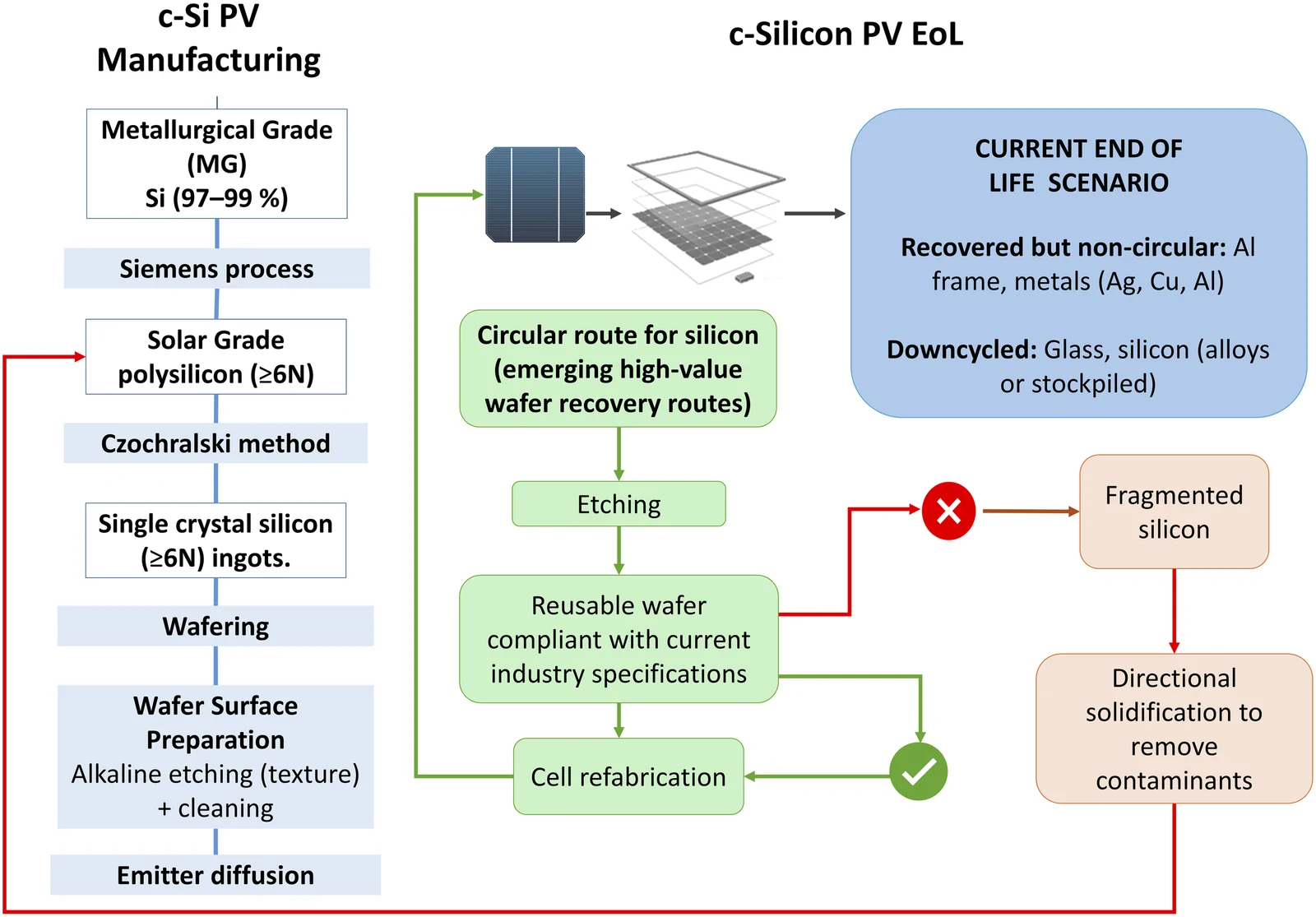
Schematic overview of crystalline silicon PV manufacturing and EoL pathways, illustrating the Siemens purification and Czochralski crystal growth process (left), current EoL route and emerging high-value wafer recovery strategies (right).

At EoL, most crystalline-silicon modules are processed in glass/metal recycling lines designed for laminated glass. Frames and junction boxes are mechanically removed, after which the laminate is shredded or thermally delaminated. The bulk of recovered mass is glass, aluminium, and copper, which are routed to commodity markets. The encapsulant-cell composite is typically incinerated or disposed of, resulting in the loss of most of the silicon and silver content.^193^ Mass flow analyses indicate that per tonne of PV waste, ≈700 kg glass, 180 kg aluminium, and 3.3 kg copper are recovered, and that the environmental benefit of the process is dominated by avoided primary aluminium and glass production rather than by cell materials.^124,193^

Although Europe's WEEE framework has enabled formal collection schemes, current treatment volumes are still modest (tens of kilotonnes per year).^193^ Life-cycle assessment (LCA) of four representative European recyclers showed that recycling contributes <1.1% of the life-cycle impact of a 3 kWp rooftop system and that, when system expansion credits are applied, the net impact becomes beneficial. The electricity demand of the mechanical lines averages ≈0.11 kWh kg^−1^, and gate fees are typically 0.17–0.25 € per kg, plus transport.^193^

#### 5.1.2 Emerging high-value routes (recovery of silicon wafers)

More selective flowsheets are now being demonstrated that recover silicon and silver in addition to glass, aluminium, and copper. The European Commission's FRELP pilot line achieved ≈98% glass, 99% Al, 95% Si, 99% Cu, and 94% Ag recovery, lowering midpoint impacts by ≈10–15% and reducing abiotic resource depletion by two orders of magnitude relative to current practice.^124^ ROSI's industrial plant in France has further shown that recovering high-purity silver, copper, and silicon can yield >90% impact reductions in 9 of 14 categories when compared with virgin material production.^195^

Because polysilicon synthesis and wafering account for 50–70% of the module's GHG footprint and between 30 and 40% of its manufacturing cost,^93^ preserving wafers at EoL offers the largest potential reduction in embedded impact. To enable wafer recovery, shredding must be omitted.^124^ The difficulty of recovering silicon wafers without damage originates in their mechanical rigidity, brittleness, and chemical inertness. The covalently bonded diamond-cubic lattice gives high modulus but low fracture toughness, making wafers prone to cracking under mechanical or thermal stress. At the same time, the strong Si–Si bonding and low chemical reactivity that confer electronic stability also prevent low-temperature dissolution or re-melting, so separation of encapsulant and silicon must rely on high heat (150–250 °C) or aggressive solvents/alkalis.^124^ To avoid wafer breakage, delamination must proceed *via* controlled thermal softening of EVA or POE encapsulants, followed by manual or mechanical separation operations that are energy- and labour-intensive.

Even when wafers are recovered intact, they require extensive reconditioning before reuse. Front-side Ag grids are stripped in nitric acid, SiN_*x*_ antireflection coatings are removed with hydrofluoric acid, and the doped emitter layer is etched back by several micrometres using HF/HNO_3_ or HF/H_2_O_2_/CH_3_COOH mixtures. The surface must then be re-textured, re-doped (POCl_3_ or BBr_3_), passivated and re-metallised.^196,197^ These multi-step wet-chemical treatments, required due to silicon's insolubility and the need to re-establish a defect-free junction, introduce acid and nitrate effluents, though LCAs confirm that such impacts are minor relative to the avoided burden of new-wafer production when recovery yields are high.^193^

#### 5.1.3 Experimental demonstrations of silicon reuse

Park *et al.*^196^ reported 16.2% cell efficiencies on reclaimed wafers, within ≈1–2% absolute of fresh controls, while Raji *et al.* achieved V_OC_ and FF comparable to those of devices fabricated with fresh wafers on reused wafers with a minority carrier lifetime of 2.7 µs and a resistivity of 1.6 Ω cm, respectively, values nearly identical to those of commercial virgin wafers.^197^

#### 5.1.4 Limitations on silicon wafer reuse: degradation of carrier lifetime and industry specification mismatches

Oxygen precipitation near the active region of the wafer is a well-documented cause of permanently low carrier lifetime, which can prevent wafer reuse. Silicon oxide precipitates and the dislocations they generate are strong recombination centres and are not dissolved by the thermal treatments normally used in cell processing.^20,21^

Voronkov and Falster further demonstrate that wafers must have a precipitate-free zone near the surfaces to maintain good electronic quality, and this zone cannot be re-established later if it was absent during crystal growth.^198^ A similar limitation arises when defects are enriched with metals. Istratov and Weber report that phosphorus-diffusion based purification removes metals dissolved in the silicon lattice but not copper, nickel, or iron that has precipitated at dislocations or precipitates, leaving lifetime irreversibly degraded.^199^ In line with these findings, wafer-recovery studies measure lifetime after layer stripping and reject wafers with persistently low values, confirming that such material states are practical limitations for reuse.^196^

Even wafers that are recovered intact and functional may not be reusable in new modules. The cell and module industry has shifted from legacy 156 × 156 mm^2^ formats to 182 and 210 mm^2^, with associated changes in stringing hardware, masks, and glass formats.^200^ Thickness windows have reduced as manufacturing moved to thinner substrates. After necessary strip and re-texturing, many reclaimed wafers fall below adequate thickness.^58^ Another factor is the evolution of the device architecture and base doping: market share has moved away from p-type boron-doped Cz for PERC toward n-type Cz for TOPCon/HJT, and, where p-type persists, gallium-doped Cz is favoured to suppress B–O LID. As a result, legacy p-type B-doped wafers may not comply with specifications for modern lines, making reuse economically unattractive.^58^

#### 5.1.5 Energy, cost, and system benefit

Silver recovery continues to be the principal economic driver of PV recycling, but wafer preservation introduces a second and potentially larger value stream that could transform recycling economics once scaled.^194^ Techno-economic reviews indicate that shredding remains cheaper on a gate-fee basis where landfill is permitted, but wafer-recovery can become competitive if yields are high and there is a market for the refurbished wafers or recovered silicon.^192^

While circularity of silicon wafers remains limited, recent improvements in the environmental impact of silicon modules have stemmed mainly from reduced material use and more efficient processes. The Siemens process achieved almost 50% energy savings over the last decade with electricity intensity declining from over 70 kWh kg in 2011 to roughly 50 kWh kg in 2021. This was a result of process efficiency gains and material efficiency innovations to reduce waste limiting actual energy consumption to 150 PJ in 2021 against a projected 430 PJ without these improvements.^72^

### 5.2 CdTe PV EoL

CdTe PV modules comprise a series of semiconductive films. This makes it difficult to delaminate and selectively remove each layer, meaning that, at EoL, the modules are either crushed and milled to facilitate leaching and recovery of the semiconductor material or disposed of in landfill (Fig. 8). However, recycling is preferred due to the rarity of the elements in CdTe modules and to avoid the accidental release of Cd, Se and Te to the environment.^120,201^

**Fig. 8:**
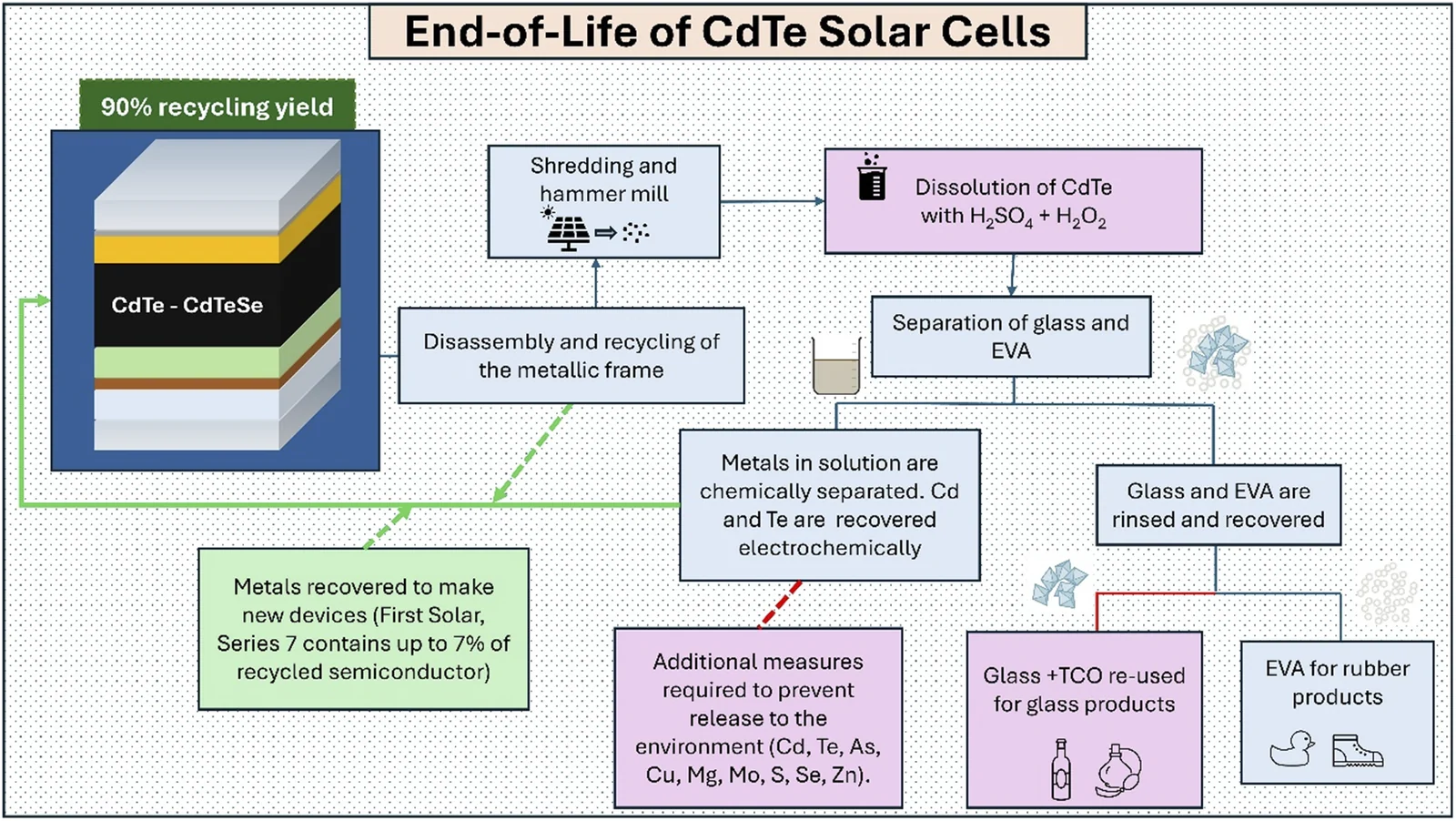
Overview of the CdTe module end-of-life recycling pathway. The EoL process includes mechanical shredding, chemical dissolution of semiconductor layers, electrochemical recovery of Cd and Te, and separation and reuse of glass, TCO, and EVA components.

The current recycling process of CdTe is mainly implemented by First Solar, where the recycling plants are integrated into the module production plants, with a current capacity of 88 000 metric tons (≈2.6 million modules) globally per year.^98,120,202^ Recycling of CdTe PV modules involves the disassembly and recycling of the metallic frame. The module is then shredded and crushed to small pieces (up to a size of ≈4–5 mm) to facilitate the ingress of leaching fluids and their separation from the encapsulant and glass components. The crushed modules are fed into a rotating drum reactor, where they are leached with a mixture of H_2_O_2_ and H_2_SO_4_ dissolving the semiconductor materials and allowing their separation from the glass and encapsulant components of the device. If the devices contain CdS, it is also dissolved at this step:^201,203,204^CdTe(s) + 3H_2_O_2_ + 3H_2_SO_4_ ⇌ CdSO_4_(aq) + Te(SO_4_)_2_(aq) + 6H_2_OCdS(s) + H_2_O_2_ + H_2_SO_4_ ⇌ CdSO_4_(aq) + S(s) + 2H_2_O

It has been reported that impurities such as Cu and other metals can be removed at this point by running the solution through a resin with a chelating agent.^202^ From this point onwards, there are two pathways for the recovery of Cd and Te. One pathway involves the use of NaOH to increase the pH up to 5.3, according to the following reaction:^204^Te^4+^ + 4NaOH(aq) ⇌ TeO_2_(s) + 2H_2_O + 4Na^+^

Te has very low solubility in acidic solutions, while Cd solubility is lower in basic solutions. This fundamental difference allows them to be separated in solution.^204^ However, the separation between Cd and Te might not be complete, and H_2_SO_4_ is employed to re-dissolve Cd in the precipitate:TeO_2_(s) + 2CdTe + 2H_2_SO_4_ ⇌ 3Te(s) + 2CdSO_4_(aq) + 2H_2_O

Te can then be separated either as TeO_2_ or Te for further refining. The remaining solution containing Cd can either be evaporated and recrystallised as hydrated CdSO_4_ containing very small amounts of impurities and then refined *via* electrowinning or it can be precipitated with CaCO_3_ (with a resulting pH between 9 and 10) to form CdCO_3_, which is then recovered and sent to a third party for further refining.^204^ Any remaining Cd and Te ions in the leached solution can be trapped with ionic resin columns to avoid their release into the environment.^201,202^

An alternative to the use of NaOH involves the use of Na_2_CO_3_ to precipitate Cd and Te:^203^CdSO_4_(aq) + Na_2_CO_3_(s) ⇌ CdCO_3_(s) + Na_2_SO_4_(aq)Te(SO_4_)_2_(aq) + 2Na_2_CO_3_(s) ⇌ TeO_2_(s) + 2Na_2_SO_4_(aq) + 2CO_2_(g)

The sediment contains Cd, S, Te and possibly other doping metals. The Te contained in the sediment is redissolved with NaOH or KOH to separate it from other metals and form soluble tellurite (K_2_TeO_3_):TeO_2_(s) + 2KOH(s) ⇌ K_2_TeO_3_(aq) + H_2_O

The sediment containing Cd is collected, while the solution with tellurite is placed in an electrolytic cell where Te^4+^ is electrowon according to the following reaction:Te^4+^ + 4e^−^ ⇌ Te^0^(s)

The metallic Te can then be recovered, while the sediment containing Cd is further processed and refined through electrochemical methods.^205^ First Solar claims to be able to recycle more than 90% of the materials (mainly glass),^98^ while for Cd and Te the leaching process has been reported to have an efficiency up to 99%, and the subsequent precipitation an efficiency greater than 99.6%.^201^ After their separation from the leaching solution the glass and encapsulant materials are washed to remove any residual traces of the leaching solution. The glass waste is resold as glass cullet, while the encapsulant material can be employed for the fabrication of rubber products.^201^

Emissions to air (<5.89 × 10^−9^ kg Cd per m^2^ of module) and water (<8.92 × 10^−8^ kg Cd per m^2^ of module) are controlled with the use of enclosed equipment, HEPA filters and on-site waste-water treatment.^120^

Although this process is already well established industrially, there have been efforts to reduce the use of wet chemical procedures. In the ANTEC Solar process, once the modules have been crushed, the material is heated and exposed to a gas mixture containing chlorine at an elevated temperature (>400 °C).^201^ The encapsulant is burnt in the process, while Cd and Te are evaporated with the gas. The chloride-rich gases are subsequently extracted and condensed as Cd- and Te-containing chlorides, from which the constituent elements can be recovered and refined.^201^ The principal attraction of this route is the elimination of aqueous processing: it avoids acidic leachates, pH-controlled precipitation, wastewater polishing and the associated need to capture residual dissolved metals. However, these benefits are exchanged for a different set of environmental and operational burdens. The process requires sustained temperatures above 400 °C, the handling of corrosive chlorine at elevated temperature, control of emissions from encapsulant combustion and robust containment of volatile cadmium- and tellurium-bearing chlorides. Neither the thermal chlorination nor the hydrometallurgical route is therefore intrinsically superior. Their relative performance depends on energy demand, recovery yield and product purity, as well as on whether a facility is better equipped to manage aqueous effluents or high-temperature, corrosive gas streams. A meaningful comparison would require both processes to be evaluated using consistent system boundaries and equivalent energy, emissions and material-recovery data.

### 5.3 Perovskite PV EoL

The solution-based processing that simplifies perovskite manufacturing also extends to EoL strategies, where low-temperature and solvent-compatible routes enable recovery approaches unavailable to silicon or CdTe technologies. Particular interest is given to the recovery of the transparent conductor-coated substrate, which accounts for approximately 40–60% of total module material cost and is estimated to account for >70% of the embodied energy, or ≈5–7 MJ per device.^206–208^ Solvent-based recovery of conductive substrates, often with one transport layer deposited on top of them, has been demonstrated without significant performance loss when reused for device fabrication, showing that remanufacture is technically feasible without cumulative damage to underlying interfaces.^206,209,210^

Early studies treated the material in the solvent effluent as potentially recoverable, while more recent studies have demonstrated actual recovery and reuse of the species it contains.^126,208^ Lead management remains a key environmental concern in closing the perovskite life cycle, making its recovery a priority. Device-level workflows report >95% Pb recovery efficiency with extracted Pb reconverted to PbI_2_ of electronic-grade purity.^126,127^ The methods most relevant to scale-up are those employing techniques with industrial precedent: ion-exchange resins, with demonstrated reuse of recovered lead iodide,^126^ and selective solvent extraction, as in the “one-key-reset” approach, in which all functional layers are recovered and reused.^211^

Routes allowing for the reuse of most of the transporting layers as recovered rather than redeposited would reduce both solvent consumption and process energy. Recent techno-economic analyses confirm that reuse of substrates with transporting layers can reduce production cost by 40–60% and embodied energy by >50% relative to building new devices, making it the most energy- and resource-efficient EoL option.^212^ These routes, however, remain limited to architectures compatible with this approach, as is the case for the printable carbon-based triple mesoporous stack, where most or all of the mesoporous stack can be reused and the degraded perovskite absorber either restored through post-treatment or removed and reinfiltrated.^213–215^

Recovery must, nonetheless, be justified against the alternative of building a device from fresh materials, which requires accounting for the energy consumption, health risk and environmental impact incurred during recovery and redeposition. Solvents are the principal liability in any large-scale projection of these methods, since several of those routinely used for perovskite layer deposition carry constraints due to their toxicity and safe handling requirements.^216^ Life-cycle assessment of laboratory-scale recovery protocols accordingly identifies solvent streams as the dominant contributor to environmental impact, which solvent recovery reduces by 56–68%, with further reductions possible if the recovered solvent is itself reused.^217^ This has prompted a shift toward greener processing: aqueous or low-toxicity solvent systems have been demonstrated to separate absorber, transport layers, electrodes and substrates while simultaneously regenerating high-purity precursors with low thermal energy investment.^126,214,218^

These results, however, are mostly reported on unencapsulated, small-area, fresh devices, and further data are needed to assess viability and environmental impact. Despite its significance for future commercial modules, remanufacturing has not broadly addressed encapsulated devices, in part because standard encapsulation protocols are not yet established for perovskites. Such protocols will likely need to satisfy two requirements simultaneously: isolation from environmental stressors and some form of lead containment^219^ in the event of module damage. The materials selected to meet these requirements will in turn determine the accessibility of the device at EoL. Two studies have demonstrated recovery through delamination: one involving epoxy-encapsulated devices after thermal treatment at 250 °C for 2 minutes,^126^ and another involving modules sealed with thermoplastic polyolefin (TPO) and a polyisobutylene (PIB)-based edge seal by mechanically assisted thermal delamination at 120–140 °C.^212^ These examples underscore that the energy requirement and methodology of delamination are contingent on the sealing materials rather than on the device itself.

On present evidence, the modest energy and temperature requirements of remanufacturing and material recovery routes available for perovskites are attractive in contrast to the high-temperature (>1000 °C) wafer purification and etching steps required for silicon. Further work is nonetheless needed on the delamination challenges introduced by specific encapsulation methods and on devices aged to failure, which would give realistic information on delamination behaviour, the state of recoverable material, recovery yields and the second-life performance of reused components.

## 6 Conclusions

Absorber chemistry links atomic-scale properties to industrial and lifecycle outcomes. The high purity requirements of silicon embed substantial energy and capital costs, but the resulting material possesses chemical inertness that underpins exceptional operational stability, with module failure arising primarily from degradation of encapsulation, metallisation, and interconnection rather than from the absorber itself. This same inertness, however, combined with the constraints of wafer geometry and silicon's chemical robustness, complicates straightforward recovery or repurposing at EoL, limiting circularity despite mature manufacturing and long operational lifetimes.

CdTe offers a contrasting balance. Thin-film processing reduces material demand and embodied energy relative to silicon while maintaining comparable operational stability. However, reliance on scarce tellurium and toxic cadmium introduces supply-risk and environmental considerations that make material recovery essential. These constraints suggest that, while CdTe represents a highly efficient and stable thin-film technology, long-term deployment at scale may ultimately depend on improved recycling and secondary supply strategies.

Perovskites present a structural contrast at every level. Their defect tolerance allows semiconductor-quality materials to be achieved through low-temperature, solution-based synthesis, reducing thermal budgets, equipment complexity, and reliance on ultra-pure feedstocks that dominate silicon's embodied impacts. In contrast to silicon, the low enthalpy of formation and ionic nature of the perovskite absorber, which contribute to operational instability, simultaneously enable selective dissolution and low-temperature remanufacturing, allowing recovery of functional substrates and precursors with minimal energy input. Lower stability therefore pairs with inherently lower barriers to circularity, which is increasingly important given regulatory and environmental pressures surrounding lead-containing materials.

These contrasting material property profiles define not only process energy and cost structures but also the scale and organisation of production. Silicon favours centralised, large-scale manufacturing suited to gigawatt-scale deployment, whereas perovskites may enable decentralised, lower-capital production, offering opportunities for regional manufacturing and improved energy access. In this context, different photovoltaic technologies may play complementary roles in the energy transition rather than representing competing solutions.

No absorber is intrinsically sustainable. Chemical robustness can extend lifetime while hindering separation; thin absorbers reduce semiconductor mass while relying on scarce or hazardous elements; and low-temperature processability can be offset by short lifetime or intensive encapsulation. Progress will depend on low-energy routes for silicon recovery, on improving perovskite stability without sacrificing circularity, and on mitigating supply risk in thin films. The task for chemical science is therefore to co-optimise performance, durability, manufacturability, resource availability and circularity rather than improve one lifecycle stage in isolation.

## Author contributions

All authors contributed to conceptualisation, writing, reviewing and editing of the manuscript. KVV, RGR and RC reviewed the literature and assembled the content. MLD supervised the overall project.

## Conflicts of interest

There are no conflicts of interest to declare.

## Supplementary Material

SC-OLF-D6SC03126A-s001

SC-OLF-D6SC03126A-s002

## Data Availability

No primary research results, software or code have been included and no new data were generated or analysed as part of this review. Supplementary information (SI): the assumptions and calculations behind the absorber material-intensity values, the methodology and data used to score the supply-risk index, the basis of the energy metrics, and a summary of laboratory-scale perovskite stability tests under IEC-type conditions. Additionally, a supplementary workbook with the full supply-risk calculations. See DOI: https://doi.org/10.1039/d6sc03126a.
