# Supplementary material for: How absorber chemistry shapes sustainability, stability and end-of-life options in silicon, CdTe and perovskite photovoltaics
Source: Chem Sci. 2026 Oct 13. Online ahead of print. doi: 10.1039/d6sc03126a (PMC13725698; doi:10.1039/d6sc03126a)
Supplement: Supplementary file 1 [file SC-OLF-D6SC03126A-s001.pdf]

Supplementary Information (SI) for Chemical Science.
This journal is © The Royal Society of Chemistry

Electronic Supplementary Information (ESI)

How Absorber Chemistry Shapes Sustainability, Stability and End-of-Life Options in Silicon, CdTe and Perovskite Photovoltaics

The supply-risk calculation workbook (SRI_calculation_workbook.xlsx) is deposited as a supplementary data file alongside this ESI. It contains the per-cell inputs, sources, quality flags and formulae behind Tables S3–S5 and Fig. 3 of the main text.

Contents: S1 material-intensity assumptions (Table S1); S2 supply-risk index methodology (Tables S2–S5); S3 basis of the energy metrics in Table 2; S4 perovskite laboratory-scale stability testing; S5 References.

S1. Material-intensity assumptions behind Table 1

Material intensities in Table 1 are only meaningful with the underlying architecture, composition, layer thickness and module efficiency stated. Intensities convert as: intensity (t GWp⁻^{1}) = ρ_layer (g cm⁻^{3}) × d_layer (μm) × w_element × 10□ / □_module (W m⁻^{2}), where w_element is the element mass fraction of the layer and η_module the module power density.

Derivation of the perovskite and CdTe intensities. Perovskite values are derived from a single stated composition, Cs₀.₀₅(FA₀.₈₃MA₀.₁₇)₀.₉₅Pb(I₀.₈₃Br₀.₁₇)₃ (M = 611 g mol⁻^{1}), 500 nm absorber, ρ = 4.2 g cm⁻^{3} and 20% module efficiency (2.1 g m⁻^{2} = 10.5 t GWp⁻^{1} of absorber), giving Pb 3.6, I 5.4, Br 0.70, Cs 0.11 t GWp⁻^{1} (FA 0.61, MA 0.09); all values fall within the independently modelled ranges of Wagner et al.[1] CdTe values are derived from the bill of materials of Wikoff et al. (43 g CdTe + 1 g CdSe per 2.52 m^2 module, ≈2.9 μm absorber) at 19% module efficiency, giving Te ≈48 and Cd ≈43 t GWp⁻^{1}.[2]

Table S1. Assumptions underlying the material-intensity values in Table 1.

**Technology**	**Architecture assumed**	**Absorber composition**	**Thickness** (µm)	**Module efficiency**	**Intensities** (t GWp⁻^{1})	**Notes / verification**
c-Si (PERC / TOPCon)	mono-PERC and n-TOPCon, 182 mm wafer	c-Si (ρ = 2.329 g cm⁻^{3});<br>B or Ga (p-type)<br>P (n-type)	140 (PERC)<br>135 (TOPCon)	• 21.5% (PERC)<br>• 22.5% (TOPCon), back-calculated for internal consistency with the intensity values	• c-Si 1499 (PERC)<br>• 1377 (TOPCon)<br>• dopants ≪1	Wafer thickness consistent with ITRPV.[3] Kerf and ingot losses excluded (≈+50% if included: 595 vs 396 g m⁻^{2})[4]
CdTe	superstrate CdSeTe/CdTe (Series 7-class)	CdTe absorber with thin CdSeₓTe₁₋ₓ graded front region (43 g CdTe + 1 g CdSe per 2.52 m^2 module; Se = 0.17 g m⁻^{2})[2]	2.9 (17.1 g m⁻^{2} CdTe, ρ = 5.85 g cm⁻^{3})[2]	19%	Te ≈48; Cd ≈43 (derived from the ref 2 bill of materials; literature range ≈38–48 for 2.0–2.9 µm absorbers)	roadmap thinning and higher material utilisation could reduce Te intensity substantially [2]
Perovskite	single-junction n-i-p, glass/glass	Cs₀.₀₅(FA₀.₈₃MA₀.₁₇)₀.₉₅Pb(I₀.₈₃Br₀.₁₇)₃<br>(M = 611 g mol⁻^{1}, ρ = 4.2 g cm⁻^{3})	0.5	20%	Pb 3.6; I 5.4; Br 0.70; Cs 0.11 (FA 0.61, MA 0.09)	derived from the stated composition; within the modelled ranges of Wagner *et* al.[1]

a

S2. Supply-risk index methodology (supports Table 3 and Fig. 3)

The supply-risk index (SRI) is a study-specific comparative indicator of the relative likelihood that access to a material becomes constrained during large-scale deployment of the PV technologies assessed. Six indicators are scored 0–10 against the thresholds in Table S2 where:

EOL-RIR, end-of-life recycling input rate: the share of total material input into the production system supplied by recycled end-of-life (post-consumer) scrap, per the European Commission criticality methodology.[5] It is distinct from the end-of-life recycling rate (EOL-RR) of UNEP (2011),[6] which is normalised to end-of-life flows rather than total supply; for fast-growing markets such as PV the two diverge strongly, and EOL-RIR is the appropriate measure of the supply relief that recycling provides today.

Concentration (indicators C and P) is scored on the top-producer share, a conservative lower bound on market concentration that requires no full country-resolved production data; Herfindahl–Hirschman-type indices provide a finer measure where such data exist, but were not needed to resolve the classes used here.

G = geological scarcity,
C = production concentration
B = by-product dependence
R = the secondary-supply deficit ($R = 10\times(1 - \text{EOL-RIR}^{1}/100)$)
D =projected PV demand pressure and
P = refining/processing concentration.

and aggregated as: $$SRI = 0.2\mathbf{G} + 0.25\mathbf{C} + 0.2\mathbf{B} + 0.15\mathbf{R} + 0.15\mathbf{D} + 0.05\mathbf{P}$$

The weighting prioritises production concentration, scarcity and by-product dependence, the mechanisms most consistently identified in the criticality literature.[7,8] Scope is global; reference year 2024/25. Scoring rule for D: each element is scored for its role in the three technologies assessed (e.g. Ga as the Cz-Si dopant, ~3% of current production), not for demand from PV technologies outside the paper's scope (e.g. III–V multijunction, whose Ga demand can exceed current production.[9] The index deliberately excludes toxicity and environmental hazard (treated separately in Table 3), substitutability (a technology-vulnerability property, discussed qualitatively in the text), economic importance and disruption consequences; values are therefore not official EU CRM supply-risk scores. Each input carries a data-quality flag (A ±0.5, B ±1, C ±2, D ±3 score units) shown as uncertainty bands in Fig. 3.

---

[1] **EOL-RIR — end-of-life recycling input rate**: the share of the total material input into the production system that comes from recycled *end-of-life* (post-consumer, "old") scrap. Denominator = all material supplied (primary + secondary); numerator = secondary material recovered from products that reached end of life. Pre-consumer/new scrap (production offcuts) doesn't count.[5]

Table S2. Scoring thresholds. G uses the qualitative rubric with depletion time (DT) as evidence only: reserve-based DT systematically mis-scores large-market metals whose reserves are economic snapshots (Pb, Zn, Cu, Sn all have DT 15–45 yr without being scarce).

Score	G: scarcity (qualitative; DT = reserves/production as evidence)	C/P: concentration (top-1 producer share)	B: by-product share	R: recycling deficit (input: EOL-RIR)	D: projected PV demand / current production
0	Very abundant	<25%	<10%	100%	<1%
2	Abundant; purity/deposit constraints	25–35%	10–25%	80%	1–5%
4	Moderate availability	>35–50%	>25–50%	60%	>5–20%
6	Scarce	>50–65%	>50–75%	40%	>20–50%
8	Very scarce / uncommon deposit types	>65–80%	>75–90%	20%	>50–100%
10	Exceptionally rare / structurally constrained	>80%	>90%	0%	>100%

Table S3. Raw input data. Production/reserves: USGS Mineral Commodity Summaries 2026 (2025 estimates)[10]; refined-stage and US-withheld values: BGS World Mineral Production 2020–2024[11]; EOL-RIR: EU CRM 2023 study, Annex 11;[5] PV demand ratios: Wagner et al.[1] Table S1, Gervais et al.[9] (Si up to ~70% in the 70 TW scenario; ~20% with circularity), with McNulty & Jowitt,[12] Ren et al.[13] and Zhou et al.[14] as corroboration. Producer shares refer to the stage scored: refinery

stage for Te, Se, Cd, Cs and In; mine/extraction stage otherwise. Per-cell source notes: deposited workbook SRI_calculation_workbook.xlsx.

Material	Production (t yr^{-1})	Reserves (t)	Top producer (share)	By-product share (%)	EOL-RIR (%)	PV demand / production (%)	Refining top-1 share (%)	Quality
Silicon	3600000		China (79%)	0	0	70	93	B
Phosphorus	250000000	73000000000	China (44%)	0	0		75	C
Boron	4130000		Türkiye (48%)	0	1		70	B
Gallium	900		China (98%)	95	0	3	98	B
Arsenic	61000		Peru (49%)	95	0		60	C
Tellurium	1000	35000	China (80%)	92	1	60	75	A
Cadmium	23000	600000	China (41%)	95	30	5	41	B
Copper	23000000	1000000000	Chile (24%)	0	55	2	48	B
Selenium	3700	92000	China (53%)	95	1	4	53	C
Zinc	13000000	240000000	China (31.6%)	0	34	0.5	47.5	C
Magnesium	982712		China (91%)	0	13	0.5	97	B
Lead	4500000	95000000	China (42%)	0	83	1	50	B
Tin	290000	6000000	China (28%)	0	31	4	52	B
Caesium	2200	200000	China (65%)	60	0	561	90	B
Methylammonium (MA)			synthetic (0%)	0	0	0	0	B
Formamidinium (FA)			synthetic (0%)	0	0	0	0	B
Bromine	665000		USA (34%)	0	0	0.58	30	C
Iodine	35000	6300000	Chile (65%)	40	0	36.5	65	B
Organic ligands / SAMs			synthetic (0%)	0	0	0	0	C

Table S4. Component scores and weighted SRI (weights: G 0.20, C 0.25, B 0.20, R 0.15, D 0.15, P 0.05).

Material	G	C	B	R	D	P	SRI	Class
Silicon	0	8	0	10	8	10	5.20	Moderate
Phosphorus	0	4	0	10	0	8	2.90	Low
Boron	1	4	0	10	0	8	3.10	Low
Gallium	5	10	10	10	2	10	7.80	High
Arsenic	4	10	10	10	0	6	7.10	High
Tellurium	8	8	10	10	10	8	9.00	Very high
Cadmium	4	4	10	7	2	4	5.35	Moderate
Copper	2	0	0	5	2	4	1.65	Very low
Selenium	7	6	10	10	2	6	7.00	High
Zinc	3	2	0	7	0	4	2.35	Low
Magnesium	0	10	0	9	0	10	4.35	Moderate
Lead	3	4	0	2	0	4	2.10	Low
Tin	6	2	0	7	2	6	3.35	Low
Caesium	9	6	6	10	10	10	8.00	High
Methylammonium (MA)	0	0	0	10	0	0	1.50	Very low
Formamidinium (FA)	0	0	0	10	0	0	1.50	Very low
Bromine	0	6	0	10	0	2	3.10	Low
Iodine	2	6	4	10	6	6	5.40	Moderate
Organic ligands / SAMs	0	0	0	10	0	0	1.50	Very low

The scores in Table S4 for C, B, R and P follow directly from the inputs in Table S3 through the thresholds in Table S2; G is scored against the qualitative rubric, with depletion time (reserves/production) as supporting evidence only, and D from the published deployment scenarios cited in Table S3. The complete calculation — every per-cell input with its source and quality flag, the indicator formulae and the weight-sensitivity analysis — is provided in the deposited workbook *SRI_calculation_workbook.xlsx*.

Weight sensitivity. Because no consensus weighting scheme exists for supply-risk indices,[7] the sensitivity of the classification to the weights was tested in two ways. (i) Equal weighting: each indicator is assigned a weight of 1/6, so the SRI reduces to the unweighted mean of the six scores. (ii) Leave-one-indicator-out: each indicator is removed in turn and the remaining weights renormalised to sum to unity, SRI₋ᵢ = (SRI − wᵢsᵢ)/(1 − wᵢ); the range across the six removals is reported. A classification is treated as robust if the risk class is unchanged across all cases. Classes are stable except at two band boundaries — Cu (Very low → Low) and Cs (High → Very high) under equal weighting; no material moves more than one band, and no High or Very-high classification depends on the weighting choice (Table S5).

Table S5. Weight sensitivity of the SRI classification; procedures defined in Section S2. Full calculation: deposited workbook SRI_calculation_workbook.xlsx.

Material	SRI (baseline)	SRI (equal weights)	SRI range (leave-one-out)	Class stable?
Silicon	5.20	6.00	4.27–6.50	yes
Phosphorus	2.90	3.67	1.65–3.62	yes
Boron	3.10	3.83	1.88–3.88	yes
Gallium	7.80	7.83	7.07–8.82	yes
Arsenic	7.10	6.67	6.13–8.35	yes
Tellurium	9.00	9.00	8.75–9.33	yes
Cadmium	5.35	5.17	4.19–5.94	yes
Copper	1.65	2.17	1.06–2.20	NO — report
Selenium	7.00	6.83	6.25–7.88	yes
Zinc	2.35	2.67	1.53–2.94	yes
Magnesium	4.35	4.83	2.47–5.44	yes
Lead	2.10	2.17	1.47–2.62	yes
Tin	3.35	3.83	2.69–4.19	yes
Caesium	8.00	8.50	7.65–8.67	NO — report
Methylammonium (MA)	1.50	1.67	0.00–2.00	yes
Formamidinium (FA)	1.50	1.67	0.00–2.00	yes
Bromine	3.10	3.00	1.88–3.88	yes
Iodine	5.40	5.67	4.59–6.25	yes
Organic ligands / SAMs	1.50	1.67	0.00–2.00	yes

S3. Basis of the energy metrics in Table 2

Energy payback is reported on two bases. System NREPBT (non-renewable primary energy) refers to a complete 3 kWp rooftop system in Europe (975 kWh kWp^{-1} yr^{-1}, 30-year panel service life[15]); no commercial perovskite system exists. Module-only EPBT (total cumulative energy demand) uses the single consistent basis of Tian et al.[16] (Southern Europe, 1,700 kWh m^{-2} yr^{-1}, performance ratio 0.75); current-vintage values for c-Si and CdTe are derived from the Table 2 embodied-energy entries at the same basis (grid efficiency 0.30). Embodied energy and embodied carbon are cradle-to-gate, per m^2 of module: total CED for c-Si;[4] total primary energy per the manufacturer's EPD for CdTe,[17] cradle-to-gate A1–A3. The 3–5-month figure quoted in the text is a manufacturing-emissions payback, not an EPBT; the 1.52-yr benchmark[18] is a US-SW system value. Payback times scale inversely with irradiation; EPBT and the per-m^2 embodied metrics are lifetime-independent — lifetime enters only the LCOE.

S4. Perovskite laboratory-scale stability testing

Table S6. Laboratory-scale perovskite devices tested against IEC qualification conditions: device specifications, test parameters, and outcomes.

Ref.	Configuration	Encapsulation	Area	Initial PCE	Tests & conditions	Result vs criterion	Lab reproduced IEC test
Mei et al. [19]	Printable mesoscopic TiO₂/ZrO₂/carbon cells, (5-AVA)ₓMA₁₋ₓPbI₃	Hot-melt PU film + glass back cover	0.8 cm^2 active, 0.1 cm^2 masked	Not in main text — their Fig. S2	DH 85 °C/85% RH, 1,100 h; TC −40↔85 °C, 200 cyc; UV 60 °C, 50 kWh m⁻^{2}; MPPT 55 ± 5 °C, 1,000 h (one cell >9,000 h)	No significant drop in all four (above their 95% line); ≤5% loss per test	IEC 61215:2016, main items
Cheacharoen et al.[20]	Cs₀.₁₇FA₀.₈₃Pb(Br₀.₁₇I₀.₈₃)₃, ITO top electrode (tandem-relevant stack), cells	Glass–glass; butyl-rubber edge seal (desiccated, 1.5 cm) + low-modulus polyolefin ("ENLIGHT", 7 MPa)	5 × 5 cm substrate; 0.12 cm^2 aperture	>11% after encapsulation, some >14%	DH 85 °C/85% RH, 1,000 h (10 cells, 5 packages); TC −40↔85 °C, 200 cyc (9 cells, 3 packages)	DH: 99% of initial PCE on average; TC: 9/9 passed with av. +3%	IEC 61646 (thin-film; DH and TC tests)
Shi et al.[21]	FTO/c-TiO₂/mp-TiO₂/CsFAMA or FAMA/PTAA/Au cells	PIB or polyolefin polymer blanket + cover glass (also PIB edge seal — failed)	~0.9 cm^2 active (9 × 10 mm)	Not in main text — their Table S4	DH 85 °C/85% RH; Humidity Freeze (50 TC prerequisite + −40↔85 °C/85% RH cycles)	PIB-blanket CsFAMA: T95 > 1,800 h DH and >75 HF cycles vs required 1,000 h / 10 cycles (<5% loss)	IEC 61215:2016 DH + HF, exceeded
Azmi et al. [22]	Inverted ITO/2PACz/3D/2D(OLAI)/C60/BCP/Ag	Glass–glass, vacuum-laminated encapsulant, butyl edge seal	Not in main text — their SM	24.3% max unencapsulated; encapsulated slightly lower (Jsc loss; ~19–22%, their Table S1)	DH 85 °C/85% RH, >1,200 h; MPPT ambient air >500 h	T95 > 1,200 h (champion); three devices 19.3 ± 0.69% after test; MPPT ~95% retained	IEC 61215:2016 damp-heat item
Emery et al. [23]	p–i–n ITO/2PACz/Cs₀.₁₅FA₀.₈₅PbI₂.₅₅Br₀.₄₅/C60/SnO₂/Cu	"COM": glass–glass vacuum lamination, polyolefin + desiccated butyl edge seal (LAB glue version failed DH)	1 cm^2 active, no mask	~15% (their Fig. 2; not stated numerically)	DH 85 °C/85% RH (ISOS-D3), 1,566 h; outdoor Berlin ISOS-O3, 10 months MPP	COM: >95% after 1,566 h DH; full retention of initial efficiency over >10 months outdoors	IEC 61215 damp-heat item + ISOS-O3 link
Wang et al. [24]	Inverted ITO/NiOₓ/CsMAFA/PCBM+C60/BCP/Cr/Au (+25 cm^2 module)	Room-temp self-crosslinked fluorosilicone (CFDP/BN composite) + cover glass, epoxy edge seal	0.1 cm^2 effective (module: 15.8 cm^2)	21.91% (target-encapsulated; control 22.02%)	DH 85 °C/85% RH, 1,000 h; TC −40↔85 °C, 220 cyc	98% retained after DH; 95% after 220 TC (both above T95)	IEC 61215 DH + TC requirements

S5 References

1 L. Wagner, J. Suo, B. Yang, D. Bogachuk, E. Gervais, R. Pietzcker, A. Gassmann and J. C. Goldschmidt, *Joule*, 2024, **8**, 1142–1160.

2 H. M. Wikoff, S. B. Reese and M. O. Reese, *Joule*, 2022, **6**, 1710–1725.

3 VDMA, *International Technology Roadmap for Photovoltaics (ITRPV) - 2023 results*, 2024.

4 V. Fthenakis and E. Leccisi, *Prog. Photovolt. Res. Appl.*, 2021, **29**, 1068–1077.

5 M. Grohol and C. Veeh, *Study on the critical raw materials for the EU 2023 - Final Report*, Publications Office of the European Union, Luxembourg, 2023.

6 T. E. Graedel, J. Allwood, J.-P. Birat, B. K. Reck, S. F. Sibley, G. Sonnemann, M. Buchert and C. Hagelüken, *Recycling Rates of Metals - A Status Report. A Report of the Working Group on Global Metal Flows to the International Resource Panel.*, UNEP, 2011.

7 B. Achzet and C. Helbig, *Resour. Policy*, 2013, **38**, 435–447.

8 C. Helbig, M. Bruckler, A. Thorenz and A. Tuma, *Resources*, 2021, **10**, 79.

9 E. Gervais, S. Shammugam, L. Friedrich and T. Schlegl, *Renew. Sustain. Energy Rev.*, 2021, **137**, 110589.

10 U. S. Geological Survey, *Mineral commodity summaries 2026*, U.S. Geological Survey, 2026.

11 N. E. Idoine, E. R. Raycraft, E. J. Evans, A. J. Mills, I. R. Shaw, I. Watkins, L. Albornoz-Parra, P. Everett, S. F. Hobbs and R. A. Shaw, *World mineral production 2020-24*, British Geological Survey, Nottingham, UK, 2026.

12 B. A. McNulty and S. M. Jowitt, *Renew. Sustain. Energy Rev.*, 2022, **168**, 112838.

13 K. Ren, X. Tang and M. Höök, *Appl. Energy*, 2021, **282**, 116148.

14 Y. Zhou, J. Li, H. Rechberger, G. Wang, S. Chen, W. Xing and P. Li, *J. Clean. Prod.*, 2020, **263**, 121599.

15 *Fact Sheet: Environmental life cycle assessment of electricity from PV systems (2022 update)*, IEA PVPS, 2022.

16 X. Tian, S. D. Stranks and F. You, *Nat. Sustain.*, 2021, **4**, 821–829.

17 P. Sinha, *Environmental Product Declaration In accordance with 14025 and EN15804 +A2 - First Solar Series 7 Photovoltaic Module*, 2023.

18 X. Tian, S. D. Stranks and F. You, *Sci. Adv.*, 2020, **6**, eabb0055.

19 A. Mei, Y. Sheng, Y. Ming, Y. Hu, Y. Rong, W. Zhang, S. Luo, G. Na, C. Tian, X. Hou, Y. Xiong, Z. Zhang, S. Liu, S. Uchida, T.-W. Kim, Y. Yuan, L. Zhang, Y. Zhou and H. Han, *Joule*, 2020, **4**, 2646–2660.

20 R. Cheacharoen, C. C. Boyd, G. F. Burkhard, T. Leijtens, J. A. Raiford, K. A. Bush, S. F. Bent and M. D. McGehee, *Sustain. Energy Fuels*, 2018, **2**, 2398–2406.

21 L. Shi, M. P. Bucknall, T. L. Young, M. Zhang, L. Hu, J. Bing, D. S. Lee, J. Kim, T. Wu, N. Takamure, D. R. McKenzie, S. Huang, M. A. Green and A. W. Y. Ho-Baillie, *Science*, 2020, **368**, eaba2412.

22 R. Azmi, E. Ugur, A. Seitkhan, F. Aljamaan, A. S. Subbiah, J. Liu, G. T. Harrison, M. I. Nugraha, M. K. Eswaran, M. Babics, Y. Chen, F. Xu, T. G. Allen, A. U. Rehman, C.-L. Wang, T. D. Anthopoulos, U. Schwingenschlögl, M. De Bastiani, E. Aydin and S. De Wolf, *Science*, 2022, **376**, 73–77.

23 Q. Emery, M. Remec, G. Paramasivam, S. Janke, J. Dagar, C. Ulbrich, R. Schlatmann, B. Stannowski, E. Unger and M. Khenkin, *ACS Appl. Mater. Interfaces*, 2022, **14**, 5159–5167.

24 T. Wang, J. Yang, Q. Cao, X. Pu, Y. Li, H. Chen, J. Zhao, Y. Zhang, X. Chen and X. Li, *Nat. Commun.*, 2023, **14**, 1342.
